# *Artemisia annua* and *A. afra* Teas, Artemisinin, and Dihydroartemisinin Differentially Regulate ROS and Fibrosis-Associated Phenotypes in Human Dermal Fibroblasts

**DOI:** 10.3390/molecules31162745

**Published:** 2026-08-07

**Authors:** Samuel Isife, Trevor Bush, Isha Medasani, Melissa Towler, Pamela Weathers

**Affiliations:** 1 Department of Biology and Biotechnology, Worcester Polytechnic Institute, 100 Institute Rd., Worcester, MA 01609, USA; soisife@wpi.edu (S.I.); trevor.bush@yale.edu (T.B.); eeyore@wpi.edu (M.T.); 2 Department of Biomedical Engineering, Worcester Polytechnic Institute, 100 Institute Rd., Worcester, MA 01609, USA; ipmedasani@wpi.edu

**Keywords:** dermal fibrosis, ROS, artemisinin, dihydroartemisinin, *Artemisia annua*, *Artemisia afra*, wound healing

## Abstract

Fibrosis is driven by persistent fibroblast activation, oxidative stress, myofibroblast differentiation, and extracellular matrix remodeling, yet available antifibrotic therapies remain limited. This study evaluated whether artemisinin (ART), dihydroartemisinin (DHA), and traditional tea infusions of *Artemisia annua* and *A. afra* differentially regulate fibrosis-associated responses in human dermal fibroblasts. Neonatal and adult human dermal fibroblasts were cultured under pre-fibrotic or TGF-β/ascorbic acid-stimulated pro-fibrotic conditions and assessed for intracellular ROS, scratch-wound closure, collagen gel contraction, fibrosis-associated gene expression, and α-SMA protein abundance. *Artemisia* teas produced greater ROS reduction than purified ART or DHA, with *A. afra* showing the strongest antioxidant effect despite lacking detectable artemisinin. DHA and *A. annua* most consistently suppressed scratch closure, while *A. afra* produced intermediate inhibition and ART was comparable to vehicle control. Collagen gel contraction was most strongly reduced by *A. annua*, with DHA and *A. afra* producing intermediate suppression. Under pro-fibrotic conditions, DHA and *A. annua* downregulated *ACTA2*, *A. annua* suppressed *COL1A1*, and DHA and *A. annua* increased matrix-remodeling *MMP* expression. Reduced levels of α-SMA confirmed the antifibrotic effects. These findings indicate that antifibrotic activity differs among artemisinin-related compounds and whole-plant *Artemisia* preparations, with DHA and *A. annua* showing the strongest overall activity.

## 1. Introduction

Fibrosis is a pathological form of wound resulting in excessive scar formation, characterized by the accumulation of extracellular matrix (ECM), persistent activation of fibroblasts, and progressive tissue remodeling that ultimately impairs normal tissue structure and function [1,2]. While scar formation is a healthy component of tissue repair, fibrosis reflects failure to resolve this process. Fibrosis represents a common pathway to organ failure across multiple systems, including the lung, liver, kidney, and skin [1,2].

**Figure 1:**
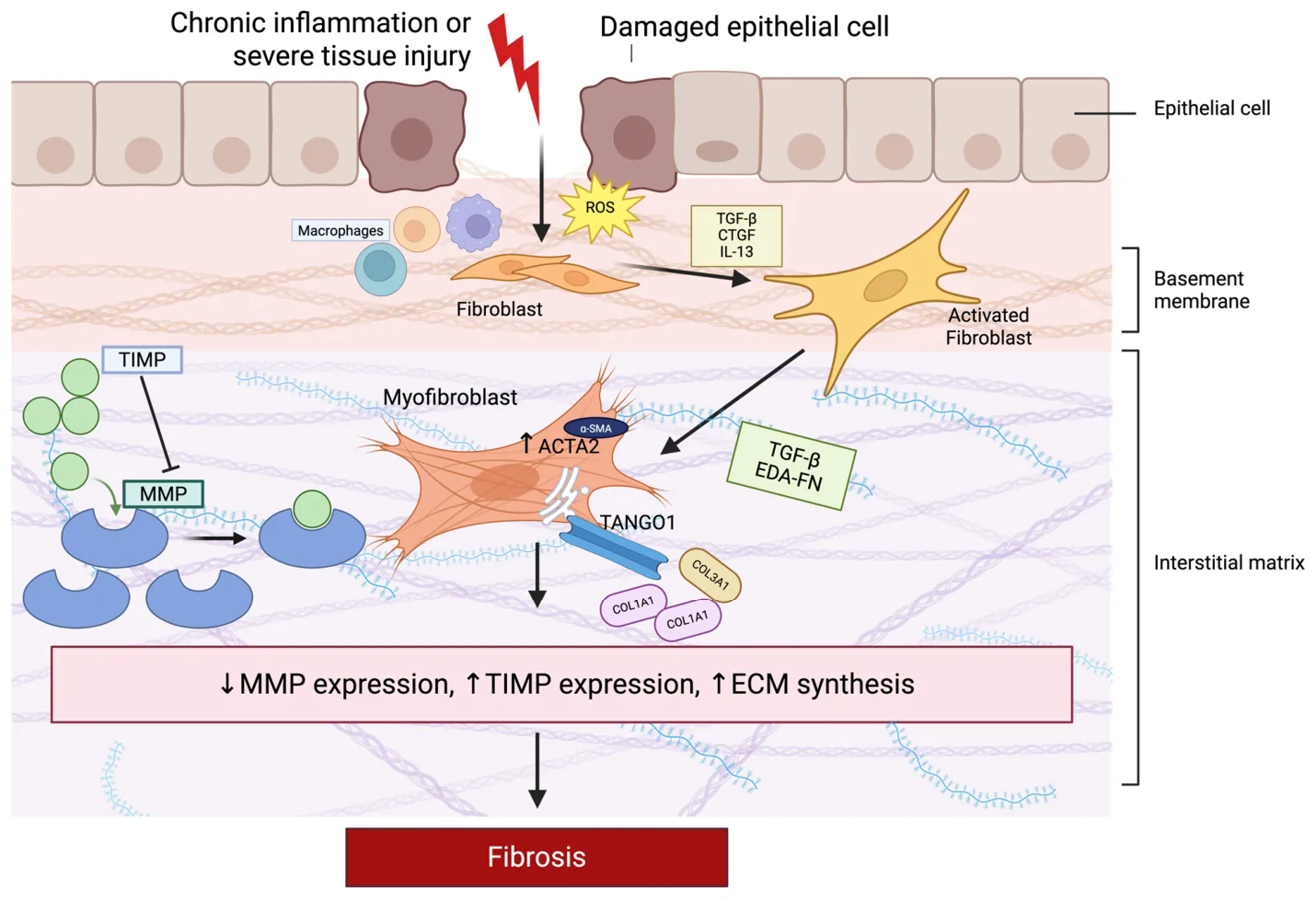
Schematic overview of the fibrotic signaling pathway in human dermal fibroblasts. Modified “Overproduction of Extracellular Matrix and Cancer Risks” by S. Isife is adapted from “Overproduction of Extracellular Matrix and Cancer Risks” created in BioRender by Eunice Huang (https://app.biorender.com/biorender-templates/details/t-64c70e20120ed9288d340cc4-over-production-of-extracellular-matrix-and-cancer-risk; accessed on 4 August 2026). Modified figure created in BioRender. Isife, S. (2026) https://BioRender.com/19g2p5e; accessed on 4 August 2026 is licensed under CC BY 4.0.

At the cellular level, reactive oxygen species (ROS) initiate fibrotic progression leading to the sustained activation of fibroblasts into matrix-producing myofibroblasts, a process strongly regulated by pro-fibrotic signaling pathways, most notably transforming growth factor beta (TGF-β) (Figure 1) [3]. Key components of the fibrotic cascade include α smooth muscle actin (α-SMA), collagen type I and III, fibronectin, matrix metalloproteinases (MMPs), and tissue inhibitors of metalloproteinases (TIMPs), which collectively regulate fibroblast activation and differentiation, ECM deposition and remodeling, and cellular processes such as migration and contractility [3,4].

In the skin specifically, dermal fibroblasts are primary effector cells in fibrotic remodeling, making them a relevant model for studying the cellular mechanisms that underlie skin fibrosis [5,6]. Despite its clinical significance, effective therapeutic options remain limited, and currently available antifibrotic agents, e.g., pirfenidone and nintedanib, primarily, slow disease progression rather than reverse established fibrosis [1,7]. This underscores the need for approaches that more directly target the cellular processes driving fibrotic remodeling, defined as the reduction in ECM buildup and scarring, leading to restored tissue flexibility.

**Figure 2:**
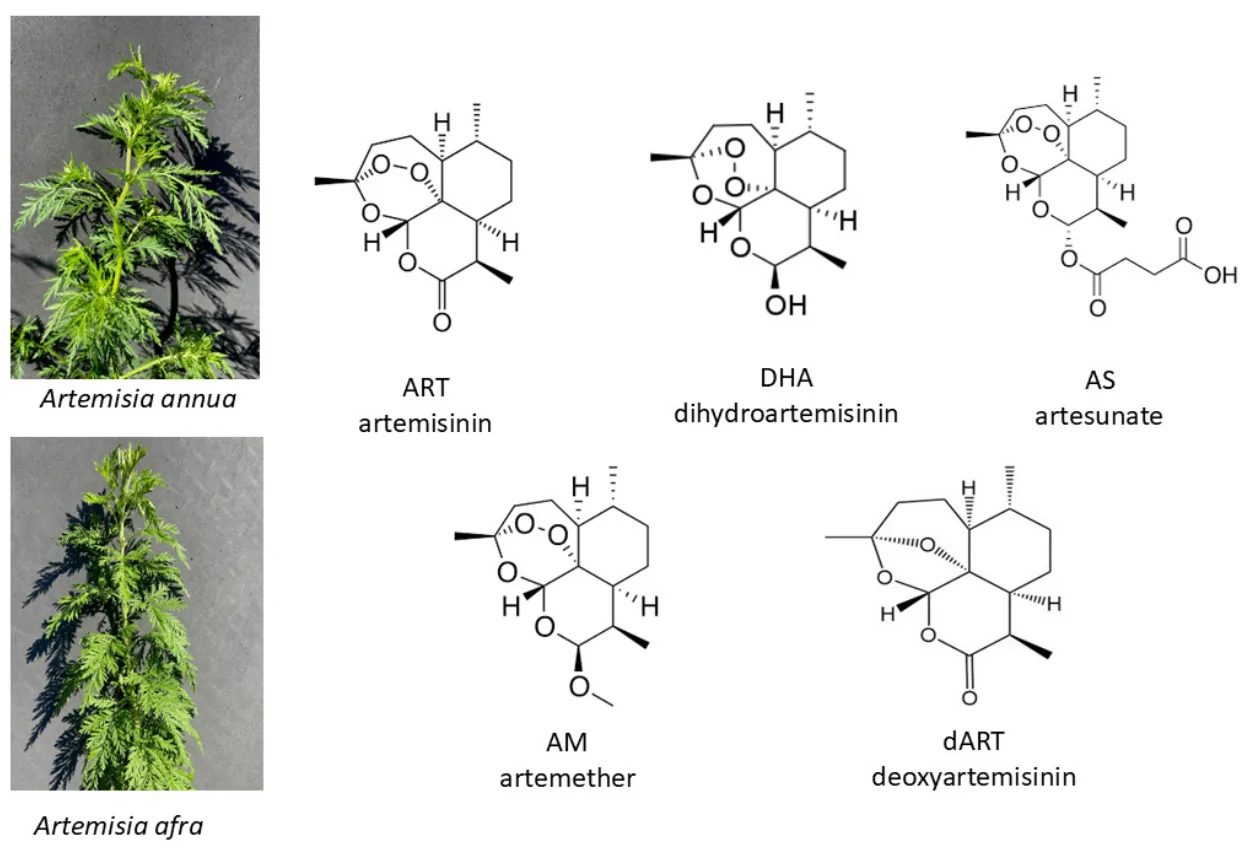
*Artemisia annua* and *A. afra* plants and artemisinic compounds artemisinin, dihydroartemisinin, artesunate, artemether, and deoxyartemisinin. Only artemisinin and deoxyartemisinin occur naturally in the plant.

Whole-plant preparations of *Artemisia annua* L. and *Artemisia afra* (Figure 2), commonly administered as tea infusions, represent accessible plant-based preparations of interest, particularly in communities where they are traditionally used and can be prepared locally [8,9]. These plants are also infused into oils and formulated as creams for dermal application. *A. annua* has a long history of medicinal use and is the source of artemisinin, a sesquiterpene lactone widely studied for its antimalarial activity and broader pharmacological effects [8,10]. While deoxyartemisinin (dART) is naturally produced by *A. annua*, it is also a liver metabolite of ART [11]. Although dART has no apparent antimalarial activity, there are reports of its antinociceptive and anti-inflammatory potency [12]. *A. afra*, a related species within the Asteraceae family, is indigenous to southern Africa, contains no detectable ART, and yet has a longstanding history of medicinal use and has shown therapeutic activity in several contexts [8,9,13]. The inclusion of both species allows us to distinguish effects that depend on artemisinin from those that may arise from the broader phytochemical composition of the plant. Prior work showed that extracts from both *Artemisia* species induced biological responses that are not fully explained by ART alone, suggesting that their activity reflects the combined contributions of multiple constituents rather than a single dominant molecule [14,15].

Reactive oxygen species (ROS) are integral to the mechanism of ART vs. the malaria parasite. ART and dihydroartemisinin (DHA) produce ROS via the iron (Fe^2+^) Fenton reaction, leading to parasite DNA damage and, subsequently, p53 activation and apoptosis [16]. TGF-β increases ROS as an effector of cell signaling, and both *A. annua* and *A. afra* are rich in flavonoids that are antioxidant phenolic compounds capable of potently scavenging ROS.

ART is normally delivered as an ART derivative (artesunate, AS; artemether, AM; dihydroartemisinin, DHA; Figure 2) to malaria patients in combination with another antimalarial drug (artemisinin drug combination therapy, ACT). Derivatization improves ART bioavailability. Although AS is frequently reported in studies describing antifibrotic activity, it is rapidly converted in vitro and in vivo to DHA [17,18], so the present study used DHA. DHA has been associated with antifibrotic and anti-proliferative effects in mammalian systems, including inhibition of fibroblast activation and modulation of pro-fibrotic signaling pathways [18,19,20]. In contrast, ART itself appears to produce more limited and context-dependent responses in fibroblast-based models [18,21]. Although ART and DHA differ by only a single functional group, a carbonyl at C10 in ART which is reduced to a hydroxyl in DHA, this substitution does not alter antimalarial efficacy, as both compounds act through the same endoperoxide-mediated, iron-catalyzed ROS mechanism that kills the parasite [22]. The C10 hydroxyl does, however, increase molecular polarity and introduces an additional hydrogen bonding capacity that may be consequential for interactions with mammalian protein targets [23]. That a structural change pharmacologically silent in one biological context produces such divergent responses in fibroblasts is a central motivation for distinguishing these compounds in the present study.

Previous studies showed that *A. afra* extract produced effects on human dermal fibroblast metabolic activity and ECM remodeling that are comparable to, and in some respects distinct from, those of *A. annua* and purified artemisinin compounds [18]. Those findings indicated that the biological activity of whole-plant preparations cannot be accounted for by ART alone and they provide the basis for the present study. Building on those results, the present study extends the analysis to both pre-fibrotic (unstimulated) and pro-fibrotic (TGF-β and ascorbic acid-stimulated) conditions. Fibroblast responses to therapeutic agents are known to depend on cellular state, yet how ART, DHA, and *Artemisia* extracts behave across these distinct fibrotic environments remain poorly defined. Existing studies have largely evaluated these agents under a single condition, leaving it unclear whether their activity is preserved, diminished, or fundamentally altered when fibroblasts are driven into an active fibrotic state by TGF-β and ascorbic acid.

Here we expand our prior study and describe how ART, DHA, and hot water infusions of a commercially validated consistent source [24] of *A. annua* and *A. afra* affect ROS production in neonatal and adult human dermal fibroblasts undergoing transition to the myofibroblast stage of fibrosis. This study also includes some key downstream effects, post ROS production. NOTE: This article is a revised and greatly expanded version of a paper entitled “Differential effects of *Artemisia* sp. and artemisinins on dermal fibrosis”, which was presented at the first International Symposium on *Artemisia*, Arusha, Tanzania, 10–12 October 2025 [25].

## 2. Results

### 2.1. Effect of Artemisia Teas and Pure Drugs on ROS Levels in Human Dermal Fibroblasts

The effects of artemisinin (ART), dihydroartemisinin (DHA), *A. annua* tea, and *A. afra* tea on intracellular ROS levels were measured in neonatal (CRL-2097) and adult (CT-1005) human dermal fibroblasts under pre- and pro-fibrotic conditions at 3 and 5 days post-seeding (DPS). ROS levels are expressed as a percentage of mean ROS normalized to the respective vehicle control (Table 1).

#### 2.1.1. *Artemisia* Teas Exert Greater ROS Reduction than ART or DHA

Across both cell lines and fibrotic conditions, *Artemisia* teas generally reduced ROS more than the purified compounds ART and DHA (Table 1). *A. afra* demonstrated the strongest ROS-lowering activity overall, producing the greatest percent ROS reduction in nearly all experimental groups. This effect was most evident at 3 DPS under pre-fibrotic conditions in adult cells, where *A. annua* and *A. afra* produced approximately 53% and 63% ROS reduction, respectively, compared with 7% and 15% reduction for ART and DHA. Similarly, in neonatal cells under pre-fibrotic conditions at 3 DPS, *A. annua* and *A. afra* reduced ROS by 25% and 35%, respectively, compared with 10% for ART and 0% for DHA. ART and DHA more modestly and broadly reduced ROS across most conditions, with no consistent evidence that either purified compound strongly suppressed ROS compared to the *Artemisia* teas.

#### 2.1.2. ROS Reduction Is Influenced by Fibrotic Condition, Cell Type, and Timepoint

The magnitude of ROS reduction was generally greatest at 3 DPS and attenuated by 5 DPS across both cell lines and fibrotic conditions, suggesting a time-dependent decline in treatment-associated ROS suppression (Table 1). Under pre-fibrotic conditions, the plant extracts reduced ROS more than under pro-fibrotic conditions, where treatment values generally shifted closer to zero, particularly at 5 DPS. Adult cells were more sensitive to *Artemisia* treatment under pre-fibrotic conditions compared to neonatal cells, with *A. annua* and *A. afra* reducing ROS by 53% and 63%, respectively, at 3 DPS in adult cells, compared with 25% and 35% in neonatal cells. Under pro-fibrotic stimulation, this cell-type difference was less pronounced, with both cell lines showing more moderate ROS reductions. Notably, *A. afra* remained more potent at ROS reduction than the purified compounds, ART and DHA, under pro-fibrotic conditions at 3 DPS in both neonatal and adult cells, supporting a consistent antioxidant effect even under fibrotic stimulation.

Because percent ROS reduction was calculated as 100 minus normalized ROS signal relative to the corresponding vehicle control, negative values indicated ROS levels above vehicle control. This was observed for DHA in neonatal pro-fibrotic cells at 5 DPS, where the value of −3% indicates a slight increase rather than a reduction in ROS relative to control.

### 2.2. Effect of Artemisia Teas and Pure Drugs on Scratch Closure in Human Dermal Fibroblasts

The effects of ART, DHA, and *A. annua* and *A. afra* teas on fibroblast migration were measured using a scratch assay in the neonatal and adult fibroblasts under pre- and pro-fibrotic conditions at 24 and 48 h post-scratch. Scratch closure was expressed as the percentage of the initial scratch area closed at each timepoint and reported as mean and statistical difference (Table 2).

#### 2.2.1. DHA and *A. annua* Consistently Suppressed Cell Migration Regardless of Condition or Cell Type

The most striking observation across all scratch assays was the consistent and profound inhibition of scratch closure by DHA and *A. annua* in both cell lines, under both pre- and pro-fibrotic conditions, and at both timepoints. Across every experimental group, both treatments kept scratch closure substantially low, in sharp contrast to ART and the vehicle control, which were less potent at inhibiting scratch closure. This pattern held with remarkable consistency regardless of cell type or fibrotic state, indicating that DHA and *A. annua* exert a robust antimigratory effect that is largely insensitive to fibrotic context or cell type.

#### 2.2.2. ART Behaved Similarly to Vehicle Control

ART produced scratch closure values statistically indistinguishable from the vehicle control in nearly all experimental conditions tested, sharing the same significance grouping across both cell lines and nearly all timepoints (Table 2). The single exception occurred in neonatal cells under pro-fibrotic stimulation at 48 h, where ART scratch closure was modestly but significantly greater than vehicle control. With this one exception, however, ART did not meaningfully impair fibroblast migration at the concentration tested, clearly distinguishing it from its derivative DHA despite their shared molecular scaffold.

#### 2.2.3. *A. afra* Showed Intermediate and Context-Dependent Inhibition

*A. afra* consistently occupied an intermediate position between the strong inhibitors, DHA and *A. annua*, and the non-inhibitory ART and vehicle control, forming its own distinct significance group in every condition tested (Table 2). Its scratch closure values were markedly more variable than the consistently low values of DHA/*A. annua* or the consistently high values of ART/vehicle. The inhibitory effect of *A. afra* was most pronounced in adult cells, particularly under pre-fibrotic conditions, where scratch closure remained well below ART and vehicle levels at both 24 h and 48 h. In neonatal cells under pro-fibrotic stimulation at 48 h, *A. afra* closure approached and statistically overlapped with both ART and vehicle control, indicating that its antimigratory activity was substantially attenuated under sustained pro-fibrotic stimulation in this cell type.

#### 2.2.4. Scratch Closure Progressed with Time, but Treatment Rankings Were Preserved

As expected, scratch closure increased from 24 h to 48 h across all treatment groups and both cell lines, reflecting ongoing cell migration over time (Table 2). Importantly, the relative ranking of treatments was largely preserved across conditions and timepoints, indicating that the antimigratory effects of DHA and *A. annua* were sustained rather than transient. The one departure from this pattern, in neonatal cells under pro-fibrotic stimulation at 48 h, suggested that this group may reflect a distinct or more variable migratory response under that specific combination of cell type, fibrotic state, and timepoint.

### 2.3. Contractility Responses

**Figure 3:**
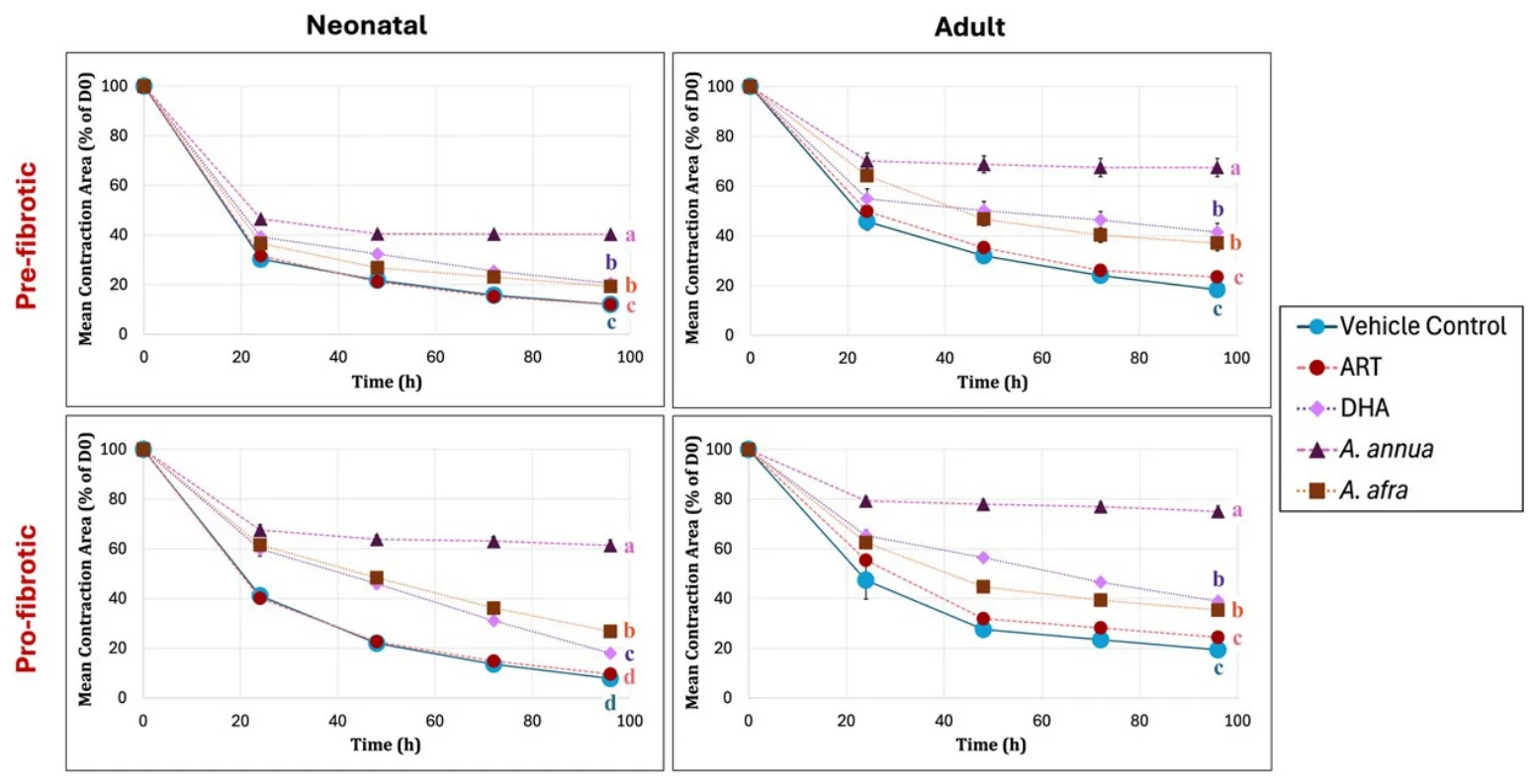
Kinetics of contraction in collagen gels of pre- and pro-fibrotic neonatal and adult fibroblasts post-drug treatment. Error bars are standard errors of the mean (SEM). Different letters indicate statistically significant differences between group endpoints within each experiment (α = 0.05). These groupings were determined by Tukey’s HSD test following an ANOVA, or by Fisher’s LSD test following a Kruskal–Wallis test.

During the first 24 h PDT, there was a rapid contraction among all treatment groups (Table 3); the rate of contraction varied substantially between mainly *A. annua* tea and the other three drug treatments. *A. annua* tea slowed contractility more than the other treatments in both pre- and pro-fibrotic conditions and in both neonatal and adult cells. After 24 h PDT, the contraction trajectories were evaluated based on changes in day 0 normalized gel area over time (Figure 3). *A. annua*-treated gels generally maintained larger gel areas throughout the remaining observation period (≥5 DPDT), indicating sustained inhibition of contraction.

#### 2.3.1. *A. annua* Strongly Inhibited the Contraction/Contractility, Pro-Fibrotic Phenotype

Contraction of the fibroblast-populated collagen matrix is a direct functional readout of myofibroblast activity. The same contractile machinery responsible for scratch closure in healing tissue becomes pathological when sustained, progressively deforming and stiffening tissue in fibrosis. Across all four contraction kinetics plots, *A. annua* consistently preserved the largest gel area, meaning fibroblasts treated with *A. annua* contracted the matrix the least of any group, never overlapping statistically with any other treatment at any timepoint or fibrotic condition (Figure 3). This indicated that *A. annua* suppresses the contractile output of activated fibroblasts irrespective of age of tissue source or fibrotic stimulus.

#### 2.3.2. DHA and *A. afra* Produced Intermediate Contraction Inhibition

DHA and *A. afra* generally formed an intermediate tier of contraction inhibition lower than *A. annua* (Figure 3). *A. afra* exhibited significantly greater inhibition of contraction than DHA only under neonatal pro-fibrotic conditions. Both treatments meaningfully reduced fibroblast-populated contraction relative to ART and the vehicle control, although neither reached the degree of inhibition achieved by *A. annua*.

#### 2.3.3. ART Did Not Inhibit Fibroblast-Populated Contraction

For both cell lines and pre- and pro-fibrotic conditions, the contraction trajectory of ART-treated collagen gels closely followed that of the vehicle control, with no marked separation between the two groups in Figure 3, indicating that ART produced little to no observable inhibition of fibroblast-populated collagen gel contraction. In contrast, the structurally related compound DHA produced greater inhibition of contraction.

#### 2.3.4. Treatment-Related Differences Persisted Across Fibrotic Conditions and Cell Lines

The treatment-related pattern described above emerged within 24 h and persisted throughout 96 h in all four contraction kinetics plots in Figure 3, indicating sustained responses by *A. annua*, DHA, and *A. afra*. The separation of these treatments from ART and the vehicle control appeared more pronounced under pro-fibrotic conditions and in adult fibroblasts.

### 2.4. Effect of Artemisia Teas, ART, and DHA on Gene Expression in Pro-Fibrotic Human Dermal Fibroblasts

**Figure 4:**
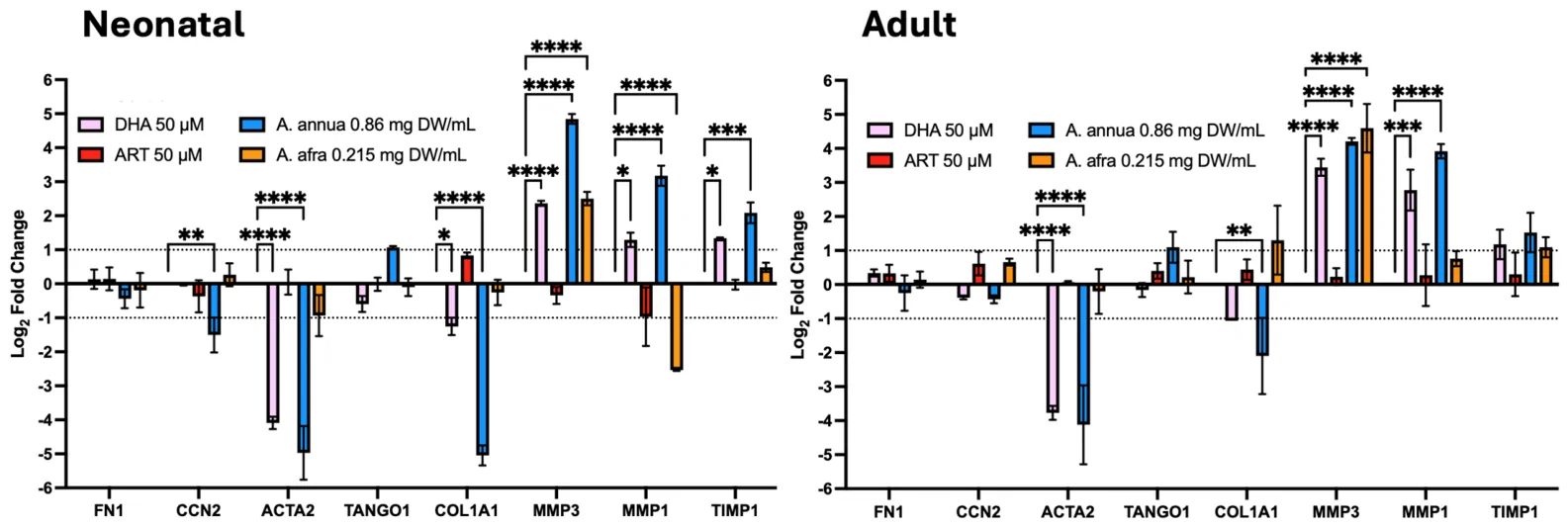
Transcription responses in pro-fibrotic cells 4 d post-drug treatment. Dunnett’s test of *n* = 2 independent experiments (3 reps/expt.); error bars are SEM compared to the solvent control that is 0. * = *p* < 0.05, ** = *p* < 0.01, *** = *p* < 0.001, **** = *p* < 0.0001.

The transcriptional responses of neonatal and adult fibroblasts to treatment under pro-fibrotic conditions were measured at 4 days post-drug treatment by measuring the log_2_ fold change in expression of key fibrosis-associated genes: *FN1*, *CCN2*, *ACTA2*, *TANGO1*, *COL1A1*, *MMP3*, *MMP1*, and *TIMP1* (Figure 4). 

#### 2.4.1. DHA and *A. annua* Consistently Suppressed Markers of Myofibroblast Activation and ECM Production

The most striking and consistent finding across both cell lines was the pronounced downregulation of *ACTA2* by DHA and *A. annua*, with log_2_ fold changes of approximately −4 to −5 in neonatal cells and −3 to −4 in adult cells (*p* < 0.0001, Figure 4). This suggested a robust suppression of myofibroblast differentiation by both treatments regardless of cell type. *COL1A1* expression was similarly reduced by *A. annua* in both cell lines (approximately −5 log_2_ fold change in neonatal HDFs, *p* < 0.0001), with DHA showing a more moderate reduction. ART and *A. afra* produced minimal or no significant changes in *ACTA2* or *COL1A1* expression, suggesting that the antifibrotic transcriptional effect is specific to DHA and *A. annua* among the treatments tested.

#### 2.4.2. Treatments Broadly Upregulated *MMP* Expression, with *MMP1* Divergent Effects

*MMP3* was significantly upregulated across multiple treatment groups in both cell lines, with DHA, *A. annua*, and *A. afra* all producing log_2_ fold changes exceeding 2 in neonatal cells and up to 4.5 in adult cells (*p* < 0.0001, Figure 4). This upregulation of *MMP3* suggests enhanced matrix degradation capacity, which could counteract ECM accumulation in the fibrotic microenvironment. *MMP1* expression showed a more divergent pattern. *A. annua* strongly upregulated *MMP1* in both cell lines, while *A. afra* produced a notable downregulation in neonatal cells (approximately −2.5 log_2_ fold change, *p* < 0.0001) but not in adult cells, pointing to a cell-type-specific effect of *A. afra* on *MMP1* regulation.

#### 2.4.3. *FN1*, *TANGO1*, *CCN2*, and *TIMP1* Show Limited Transcriptional Response

*FN1* and *TANGO1* expression remained largely unchanged across all treatment groups in both cell lines, suggesting that these ECM-related genes were not primary transcriptional targets of the treatments at this timepoint. *CCN2* showed modest downregulation with *A*. *annua* in neonatal cells (*p* < 0.01) but not in adult cells, indicating a potentially cell-type-specific effect. *TIMP1* expression was modestly upregulated by DHA and *A. annua* in neonatal cells (*p* < 0.001), but showed minimal change in adult cells, suggesting that the *TIMP1* transcriptional response may be more prominent in neonatal fibroblasts under these conditions.

### 2.5. Translational Response to Drug Treatments

**Figure 5:**
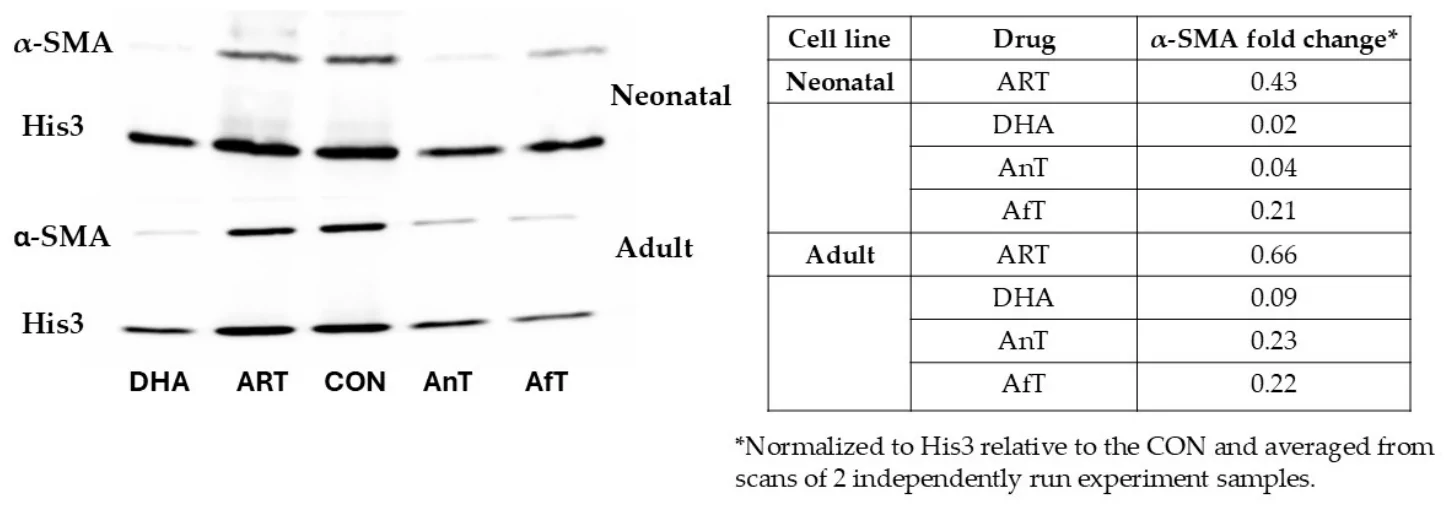
Western blot analysis of effects of ART, DHA, and *A. annua* and *A. afra* teas on α smooth muscle actin (α-SMA). Blots were from a replicated protein isolated from the same extracts analyzed using qRT-PCR. ART, artemisinin; DHA, dihydroartemisinin; CON, vehicle control; AnT, *A. annua* tea; AfT, *A. afra* tea. Fold changes in α-SMA normalized to Histone H3 (His3) relative to the vehicle control (CON) were averaged from scans of samples from two independently run experiments.

Using the same samples that were obtained from the experiments for qRT-PCR analysis of pro-fibrotic cells, protein was also extracted and analyzed to determine if there was translation of the transcripts. Figure 5 shows representative blots of *ACTA2* protein (α-SMA) after 4 d of drug treatments. ART provoked no major response, but DHA, *A. annua*, and *A. afra* reduced the amount of α-SMA, consistent with the decreases noted in the transcripts in both cell lines (Figure 4).

## 3. Discussion

Fibrosis is a pathological healing process characterized by the transdifferentiation of resident fibroblasts into highly contractile myofibroblasts, that deposit excessive collagen-rich extracellular matrix (ECM) that lacks the functional properties of uninjured tissue [1,2]. This process affects virtually every organ system and is estimated to contribute to nearly half of all deaths in the developed world, yet currently available therapies mainly slow disease progression rather than reverse established ECM accumulation [7,26]. A treatment with meaningful antifibrotic potential may need to do more than suppress fibroblast activation; it should also shift the fibrotic microenvironment toward matrix remodeling. Against this backdrop, ART-derived compounds and whole-plant *Artemisia* preparations have attracted growing interest as candidate antifibrotic agents, with reported activity in dermal fibroblasts and other organ-fibrosis models [18,21,27]. The present study evaluated the effects of ART, DHA, and whole-plant tea infusions of *A. annua* and *A. afra* on human dermal fibroblasts across ROS production, migration, fibrosis-associated gene expression, fibroblast contractile phenotype, and α-SMA protein abundance under both pre-fibrotic and pro-fibrotic conditions. Four major patterns emerged: *Artemisia* teas reduced ROS more than purified ART or DHA; DHA and *A. annua* consistently inhibited fibroblast migration and suppressed markers of myofibroblast activation; *A. annua* consistently suppressed fibroblast contractility more than the other three drug treatments; and ART alone showed limited activity across most assays. These results support but also meaningfully refine prior reports that ART-related compounds have antifibrotic activity, and they challenge several interpretive conventions in that literature.

A central finding was that *Artemisia* teas suppressed ROS more than ART or DHA, with *A. afra* showing the strongest antioxidant response despite being used at one-quarter the dry-weight concentration of *A. annua* and lacking detectable ART. This supports the view that antioxidant activity in *Artemisia* teas is not primarily dependent on ART content but reflects the broader phytochemical composition of the extracts, including phenolics, flavonoids, and other redox-active constituents. Prior surveys of *A. afra* have documented a substantial variation in phytochemical profiles across African samples [28], while the broader *Artemisia* literature supports the contribution of phenolics, flavonoids, and other secondary metabolites to biological activity but not related to fibrosis [13]. Prior studies of either *Artemisia* species to our knowledge have not used commercially available plant material that has demonstrated product consistency and [24] standardized production of the tea infusions as documented in our methods. Fibrotic state also modulated ROS responses: suppression was stronger under pre-fibrotic conditions and diminished under pro-fibrotic conditions (TGF-β/ascorbate stimulation), likely reflecting the potency of the TGF-β-driven fibrotic program in overriding treatment-induced redox changes [3]. This has a practical implication wherein antioxidant screening alone may overestimate antifibrotic potential unless functional and transcriptional endpoints are also evaluated.

This point is illustrated directly by *A. afra*, which produced the strongest reduction in intracellular ROS but showed only intermediate effects across the other fibrosis-associated endpoints. Conversely, DHA and *A. annua* demonstrated broader antifibrotic activity despite limited or more moderate ROS reduction. This disconnect indicates that ROS reduction alone is insufficient to predict antifibrotic efficacy. Fibrosis is governed by multiple cellular processes, including myofibroblast differentiation, extracellular matrix synthesis and remodeling, migration, and contractility. Strong suppression of one component, such as intracellular ROS, may therefore occur without equivalent modulation of the broader fibrotic phenotype. Plant-extract studies often treat antioxidant and antifibrotic activity as equivalent [29,30], but the present data argue against that simplification. The disconnect between *A. afra*’s ROS suppression and its modest transcriptional effects suggests that distinct phytochemical constituents are responsible for these different endpoints.

The scratch migration assay revealed a complementary pattern. DHA and *A. annua* strongly and consistently inhibited scratch closure (fibroblast migration) to below 30% of controls across both cell lines, both fibrotic conditions, and both timepoints. These findings agree with prior reports that DHA inhibits cell migration. Guo et al. showed DHA reduced VEGF-induced endothelial cell migration [31] and later identified the TGF-β1/ALK5/SMAD2 axis as a mediator of DHA antimigratory effects [32]. Artesunate (AS), which functionally acts as DHA [33] also inhibited migration of fibroblast-like synoviocytes via AKT/RSK2 suppression [34]. The present data extend this picture to pro-fibrotic dermal fibroblasts while adding a critical negative result; ART showed no meaningful antimigratory activity at the same concentration. This supports the suggestion that the C10 hydroxyl, irrelevant to antimalarial activity [23], is a structural determinant of DHA’s antifibrotic activity and migration inhibition.

The treatment-dependent differences in collagen gel contraction were consistent with the prior literature identifying fibroblast-mediated collagen gel contraction as a functional readout of fibroblast–extracellular matrix-remodeling relevant to wound healing and fibrosis [35]. In this study, *A. annua* tea most consistently inhibited collagen gel contraction, while DHA and *A. afra* tea produced intermediate inhibition, and ART behaved like the vehicle control. These findings align with our prior report that DHA and *Artemisia* tea treatments modulate fibrosis-related responses in neonatal dermal fibroblasts, including changes in *ACTA2*/α-SMA and matrix remodeling markers, whereas ART was ineffective [18]. The DHA response is consistent with results showing reduced skin fibrosis in preclinical models [36]. 

This compound-specificity challenges a persistent framing in the fibrosis literature. Much prior in vitro evidence for ART antifibrotic activity has been generated using AS or DHA, yet findings are routinely attributed to “artemisinin compounds” broadly. Larson et al. showed AS antagonizes TGF-β1-mediated myofibroblast formation in human dermal fibroblasts [21], but AS is rapidly hydrolyzed to DHA in cell culture media [18,33], meaning those effects may be substantially DHA-mediated. Wang et al. reviewed ART derivatives in fibrosis without systematically separating ART from its active metabolites [27]. The present data directly address this gap and support the argument by Weathers et al. [18] that much of the ART antifibrotic literature may describe DHA activity regardless of which compound was nominally administered. Independently, an unbiased high-throughput screen of ~5000 compounds in iPSC-derived cardiac fibroblasts identified AS, not ART, as a top antifibrotic hit acting through MD2/TLR4-ERK-AP1 suppression [37], providing orthogonal support for the compound-specificity we observed. It is also worth noting that DHA’s effects on fibroblast migration are context-dependent. Wei et al. showed DHA promoted migration in chronic skin ulcers via NFIC/FBN1 upregulation [38], opposite to what we observed in pro-fibrotic dermal fibroblasts. These findings are reconcilable because, in wound healing, enhanced migration supports repair, but, in fibrosis, its suppression limits ECM accumulation thereby, reducing scarring. This distinction has direct implications for which therapeutic context these compounds should be considered.

The transcriptional analysis under pro-fibrotic conditions further supports the antifibrotic potential of DHA and *A. annua*. Both strongly downregulated *ACTA2* with log_2_ fold changes of approximately −3 to −5 across both cell lines, while *A. annua* also strongly suppressed *COL1A1*, increasing *MMP1* and *MMP3* expression. These findings are consistent with Larson et al. [21] and with Li et al. [36], who demonstrated DHA-mediated suppression of *COL1A1* and *ACTA2* through PI3K/AKT/autophagy signaling in bleomycin-induced skin fibrosis, a pathway not tested in the present study [36]. Regarding upstream mechanisms, Chakraborty et al. identified STAT3 as a central convergence point for multiple pro-fibrotic signals in human dermal fibroblasts. STAT3 inhibition suppressed *ACTA2* and *COL1A1* in a pattern resembling the DHA response observed here [39]. Given that AS inhibits STAT3 phosphorylation and directly binds p53 in lung fibroblasts and in vivo lung tissue [40], and that DHA coordinately suppresses JAK2 and STAT3 phosphorylation in whole lung tissue from a rat pulmonary fibrosis model [41], the STAT3 axis represents a plausible but mechanistically unresolved target in dermal fibroblasts. Neither study establishes the precise interaction between artemisinin-class compounds and that pathway at the fibroblast level in skin, and future work should employ targeted pathway inhibitors or molecular docking against JAK2, STAT3, and p53 to determine where DHA acts directly, alongside PI3K/AKT and TGF-β/SMAD pathways [39,40]. Western blot data confirmed protein-level suppression of α-SMA by DHA, *A. annua*, and *A. afra*, while ART showed little response, validating the transcriptional findings. Interestingly, *A. afra* reduced α-SMA despite limited mRNA suppression, suggesting post-transcriptional or protein stability-related regulation that warrants further investigation. Several transcriptional responses differed between the neonatal and adult fibroblast lines. Most notably, *A. afra* significantly downregulated *MMP1* in neonatal fibroblasts but produced no change in adult fibroblasts. Similarly, *A. annua* modestly reduced *CCN2* expression only in neonatal fibroblasts, whereas *TIMP1* was modestly upregulated by DHA and *A. annua* in neonatal but not adult cells. These differences are consistent with reports that neonatal and adult dermal fibroblasts can differ in extracellular matrix regulation, matrix-remodeling programs, and transcriptional responsiveness [40,41]. Moreover, the neonatal and adult fibroblasts are distinct donor-derived cell lines; therefore, donor-specific genetic or epigenetic variation cannot be excluded as a contributor to the observed differences. We therefore interpret these cell-type-specific patterns as hypothesis-generating and recommend validation using multiple independent donors. *MMP3* was broadly upregulated by DHA, *A. annua*, and *A. afra*, consistent with prior dermal fibroblast data [18,21]. Combined with *ACTA2* and *COL1A1* downregulation, this suggests a coordinated shift toward ECM remodeling [6]. Remodeling encompasses the dual action required for meaningful antifibrotic activity. However, since TGF-β/SMAD3 suppressed *MMP3* in fibroblasts [3], this upregulation may partly reflect relief of TGF-β repression rather than direct induction, a distinction important for interpreting the durability of the response.

One broader implication is that whole-plant *Artemisia* preparations act through mechanisms that cannot be reduced solely to ART content. *A. annua* outperformed ART despite containing it, and *A. afra* was active without any detectable ART, suggesting an ART-independent mechanism consistent with the literature showing that *A. annua* preparations outperform pure ART through enhanced bioavailability and synergistic phytochemical activity [25]. Limitations include the use of two-dimensional culture, which does not reproduce the three-dimensional architecture or multicellular context of fibrotic tissue in vivo. Most in vivo ART antifibrotic studies used bleomycin-induced fibrosis models where fibrosis develops rapidly in continuously injured tissue [36,42]. That involves a mechanism different from slower human dermal fibrosis, and therefore in vivo validation in skin-specific models is necessary. Furthermore, in this study, the composition of the teas focused only on the target molecule ART and the chemical composition of the teas was not fully resolved, limiting attribution of activity to specific constituents. Future work should include chemical fractionation, pathway-specific inhibitor experiments, and comparison across a broader panel of donors. Furthermore, only one dose was studied, so a dose–response investigation is still warranted. 

## 4. Materials and Methods

### 4.1. Cell Cultures and Their Maintenance

Human dermal fibroblast (HDF) primary cell lines CRL-2097 (neonatal male) were from ATCC (Manassas, VA, USA). CT-1005 human female dermal fibroblasts were obtained from the University of Massachusetts’ Medical School tissue distribution program (Worcester, MA, USA), derived from the dermis of a human female patient undergoing a panniculectomy. Both lines were stable through passage 11 and experiments were conducted within those passage limits. T flasks were seeded at 5 × 10^3^ cells/cm^2^ unless otherwise noted and grown in 1× DMEM/F12+GlutaMAX^TM^ (Gibco 10565-018, Waltham, MA, USA), 10% *v*/*v* Fetal Clone III (FCIII; Cytiva SH30109.03, Malborough, MA, USA), 1% *v*/*v* penicillin–streptomycin (Gibco 15140-122, Waltham, MA, USA) and incubated at 37 °C and 5% CO_2_.

### 4.2. Plant Material

*Artemisia annua* L. cv. SAM (voucher MASS 317314) dried leaves containing about 1% (*w*/*w*) ART and *A. afra* cv. MAL (voucher FTG181107) were used. Both species were sourced from Atelier Temenos, Homestead, FL, USA, and prepared as tea infusions of 10 g dried leaves/L for these experiments. Preparation is detailed in Kane et al. [43]. ART content in the *A. annua* tea was analyzed by the gas chromatography mass spectrometry (GCMS) method described in Martini et al. [44] and was diluted to 50 µM for experiments. Representative GCMS chromatograms of *A. annua* and *A. afra* dichloromethane extracts are shown in Supplemental Figure S1. Extracts of both plants were also analyzed for ART and other phytochemicals using thin layer chromatography (TLC) as detailed in Supplemental Figures S2 and S3. There is no detectable ART in *A. afra* MAL as shown in the GCMS and TLC chromatograms.

### 4.3. Experimental Design Defining Pre- and Pro-Fibrotic Culture Conditions and Analysis Times

**Figure 6:**
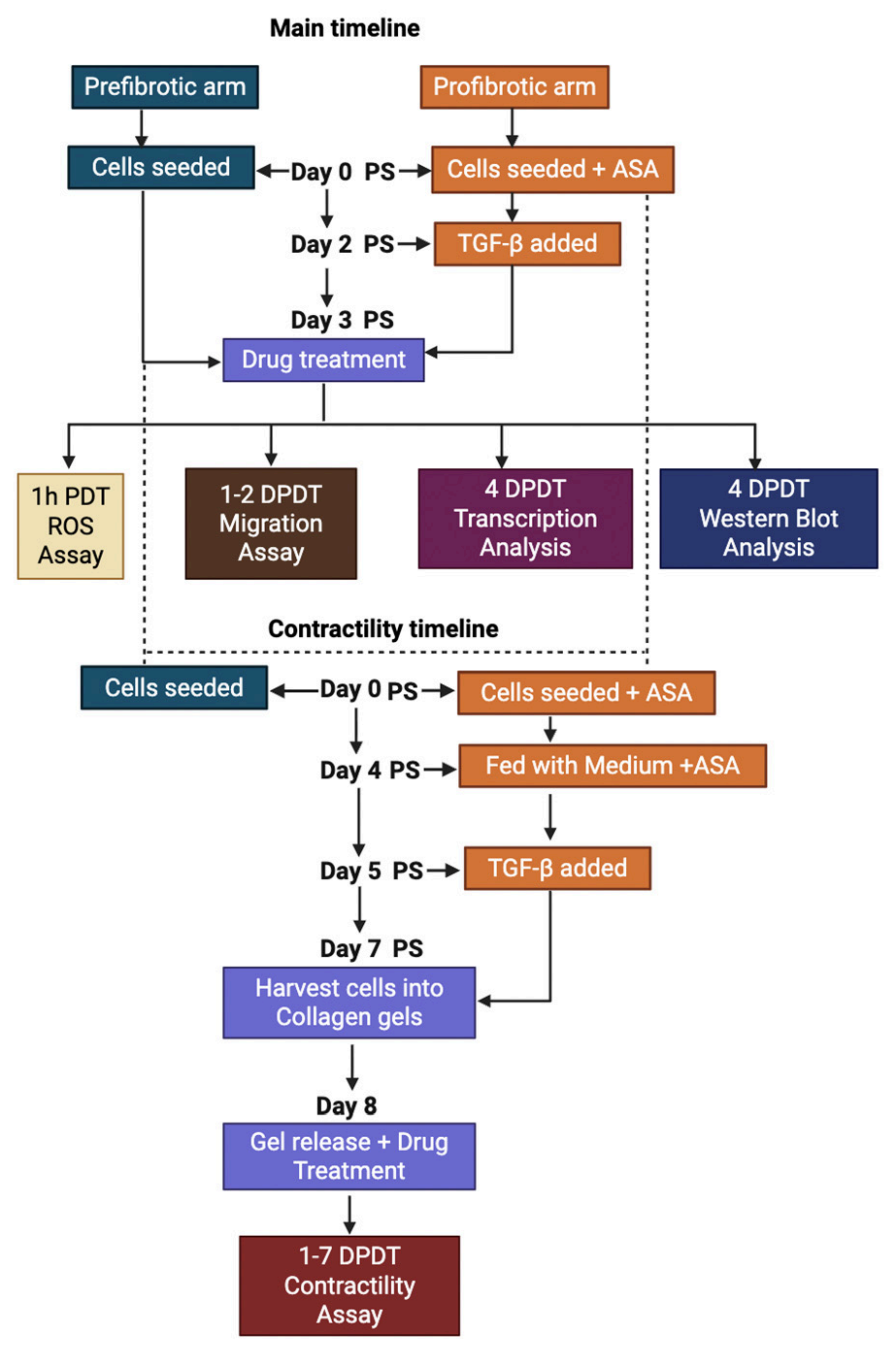
Experimental plan showing how and when pre- and pro-fibrotic cells were treated with drugs and then analyzed. DPDT, days post-drug treatment; PS, post-seeding. Created in BioRender. Isife, S. (2026) https://BioRender.com/3e0bgxd; accessed on 4 August 2026.

Fibroblasts were cultured under two experimental conditions, pre-fibrotic and pro-fibrotic, as summarized in Figure 6. For the pre-fibrotic condition, cells were seeded on Day 0 and maintained under standard culture conditions until Day 3, or Day 7 for contractility experiments, when drug treatments began. For the pro-fibrotic condition, cells were seeded on Day 0 in medium supplemented with 200 µM L-ascorbic acid 2-phosphate sesquimagnesium salt hydrate (ASA; Sigma Aldrich A8960, St. Louis, MO, USA). For all experiments except contractility, two days post-seeding (Day 2 PS), without replacing media, a stock solution of 10 µg/mL TGF-*β*1 (Cell Signaling Technology 75362S, Danvers, MA, USA) was added to each flask to a final concentration of 10 ng/mL. The following day, media was removed, and drug treatments were added. Experiments were initiated and/or performed on day 3. For ROS experiments, separate treatment experiments were performed at 3 and 5 DPS after 1 h drug treatment. For scratch-wound experiments, scratches were created on Day 3 PS, drug treatments were added immediately after scratching, and images were captured at 0, 24, and 48 h. For contractility experiments, cells were fed with fresh ASA-supplemented medium on Day 4 PS and TGF-*β*1 was added on Day 5 PS without replacing the media. Under both pre- and pro-fibrotic conditions, cells were maintained through Day 7 and then harvested for gel preparation. Experiments were initiated and/or performed on Day 3, unless otherwise specified. Contractility measurements were analyzed 1–7 DPDT. For quantitative real-time PCR (qRT-PCR), drug treatments were applied on Day 3 PS, and cells were harvested on Day 7 (4 DPDT; 7 DPS) for RNA extraction and Western analysis.

### 4.4. Drug Treatments

All drug treatments included the same amount of solvent as in the vehicle control (0.1% *v*/*v* DMSO (Sigma Aldrich D8418, St. Louis, MO, USA) and 8.6% *v*/*v* water), and concentrations were as follows: ART 50 µM; DHA 50 µM; *A. annua* tea infusion 0.86 mg DW/mL; *A. afra* tea infusion 0.215 mg DW/mL. ART (Cayman Chemical 11816, Ann Arbo, MI, USA) and DHA (TCI. America D3793, Portland, OR, USA) were dissolved in DMSO at a stock concentration of 50 mM and filter sterilized, while *A. annua* and *A. afra* solutions were 10 g DW/L tea infusions prepared as described in [43]. *A. afra* was diluted to a quarter of the concentration of *A. annua* (0.215 mg DW/mL vs 0.86 mg DW/mL), a selection informed by metabolic activity data obtained from resazurin assays [18]. At full and half strength, *A. afra* produced a notable reduction in cellular metabolic activity, suggesting that these concentrations exceeded the optimal biological range for the cells. At quarter strength, however, cellular metabolic activity was maintained at levels comparable to those observed with full strength *A. annua*, indicating that this concentration preserved cell health while retaining biological activity. This *A. afra* concentration was therefore selected for all subsequent experiments. Drug treatment media also contained 1% *v*/*v* penicillin–streptomycin, plus 200 µM ascorbic acid (ASA; for pro-fibrotic cells only).

### 4.5. ROS Assay

Intracellular ROS levels were measured using the 2′,7′-dichlorofluorescein diacetate (DCFH-DA) assay. Human dermal fibroblasts were seeded in 24-well plates at a density of 5 × 10^3^ cells/cm^2^ and cultured under either pre- or pro-fibrotic conditions as previously described. At 3 and 5 DPS, cells were washed once with phosphate-buffered saline (PBS) and incubated with DCFH-DA (Sigma-Aldrich, St. Louis, MO, USA; Cat. No. D6883) dye in Dulbecco’s phosphate-buffered saline (DPBS, Gibco 14190-136, Waltham, MA, USA) for 45 min at 37 °C in the dark. Following dye loading, cells were washed with PBS and treated with ART, DHA, *A. annua* tea, *A. afra* tea, or vehicle control for 1 h, after which intracellular ROS levels were measured by fluorescence using excitation and emission wavelengths of 485 and 520 nm, respectively, in a Perkin Elmer Victor NIVO plate reader. In parallel, fibroblasts from the same seeding were plated identically in a separate plate and, at the same 3 or 5 day PS timepoints, treated with ART, DHA, *A. annua* tea, *A. afra* tea, or vehicle control for 1 h, followed by incubation with resazurin solution (0.15 mg/mL resazurin sodium salt (Thermo Fisher Scientific B21187.03, Waltham, MA, USA) dissolved in DPBS mixed in a ratio of 2:10 with DMEM/F12+GlutaMAX^TM^) for 2 h to provide a measure of relative cell number via metabolic activity for each treatment condition. ROS fluorescence values were normalized to the corresponding resazurin fluorescence values for each treatment to account for differences in cell number. Normalized ROS levels were expressed as relative fluorescence units (RFU) compared to vehicle control. All experiments were performed as 3 independently run experiments, in which each also had 3–4 internal replicates per drug treatment condition. Data were statistically analyzed as later detailed.

### 4.6. Scratch-Wound Migration Assay

Fibroblast migration was assessed using a scratch-wound assay. Human dermal fibroblasts were seeded in 12-well plates at a density of 1 × 10^4^ cells/cm^2^ and cultured under either pre-fibrotic or pro-fibrotic conditions as described above. On Day 3 PS, cells were incubated with mitomycin C (1.25 µg/mL; Focus Biomolecules, Plymouth Meeting, PA, USA; Cat. No. 10-1170) for 30 min to inhibit cell proliferation. The mitomycin C concentration and exposure time were selected following a dose–response and time-course optimization (1.25–10 µg/mL; 30 min–2 h), in which higher concentrations and/or longer exposure times resulted in cell death. A concentration of 1.25 µg/mL for 30 min was identified as the minimum effective condition that arrested proliferation without compromising viability, confirmed by slower wound closure in mitomycin C-treated wells relative to untreated controls. A linear scratch was created in each well using a sterile 1000 µL pipette tip, and detached cells were removed by washing with DPBS. Cells were then treated with ART, DHA, *A annua* tea, *A. afra* tea, and a vehicle control in medium containing 1% serum. Images of the wound area were captured immediately after scratching (0 h) and at 24 and 48 h using phase-contrast microscopy. Scratch closure was quantified using ImageJ/FIJI version 1.54p with a wound healing plugin macro (updated version) (https://github.com/AlejandraArnedo/Wound-healing-size-tool/wiki; accessed on 7 December 2025), by measuring the scratch area at each timepoint. Migration was expressed as percentage scratch closure relative to the initial scratch area at 0 h. All experiments were performed with two independent biological replicates, each with four technical replicates, and data were statistically analyzed as later detailed. 

### 4.7. Contractility Assay

Fibroblasts were seeded in T flasks at 6.7 × 10^3^ and 5 × 10^3^ cells/cm^2^ for pre- and pro-fibrotic conditions respectively. Media was refreshed at 4 d and passaged at 7 d post-seeding for pre- and pro-fibrotic conditions as already described. Prior to resuspension pro-fibrotic cells were sieved through a sterile 70 µm cell strainer (Greiner Bio-One 542070, Monroe, NC, USA) to obtain a uniform single-cell suspension. This step was performed because increased extracellular matrix-related expression under pro-fibrotic culture conditions (ASA and TGF-β) may promote cell clustering [45]. Fibroblast-populated collagen gels were prepared using a modified method adapted from [46] and maintained under pre- or pro-fibrotic conditions as appropriate. Gels containing 2.5 × 10^4^ cells were formed in non-tissue-culture-treated 12-well plates (area = 3.8 cm^2^, *n* = 3 per condition), with reagent volumes scaled proportionally to the original 6-well plate protocol, and were released approximately 24 h after preparation. Drug treatments were administered as previously described. Gel contraction was monitored by daily imaging for four days after gel release. Gel area was quantified using ImageJ/FIJI following spatial calibration with a 0.4 cm reference grid.

### 4.8. Transcription Analysis

After 4 d of drug treatment, RNA was extracted from cells using the NucleoSpin RNA/protein mini kit (Macherey-Nagel 740933.50, Bethlehem, PA, USA) per manufacturer instructions. RNA was measured using a NanoDrop™. CRL-2097 cDNA was synthesized using Thermo Fisher High-Capacity cDNA Reverse Transcription Kit (4367714, Waltham, MA, USA) according to the manufacturer’s instructions, and CT-1005 cDNA was synthesized using either the Thermo Scientific Verso cDNA Synthesis Kit (AB1453A) or the Thermo Fisher High Capacity cDNA Reverse Transcription Kit (4367714, Waltham, MA, USA) (replicate experiments 1 and 2, respectively). The cDNA synthesis kit choice did not affect outcomes, as verified by the consistency of the resulting data in replicated experiments. SYBR-based PowerTrack master mix (Thermo Fisher Scientific A46109, Waltham, MA, USA) was used for qRT-PCR on a Thermo Fisher Scientific QuantStudio 6 Pro Real-Time PCR system in 10 μL reactions using the following thermal protocol: 95 °C for 2 min, then 40 cycles of 95 °C for 15 s and 60 °C for 60 s. Primer sequences are listed in Table 4; all were purchased from Eton Biosciences, Newton, MA, USA Fold change in gene expression was determined using the ΔΔCT method [47,48] with *GAPDH* as housekeeping gene. Up or downregulation was defined as exceeding a twofold change in transcription. Each experiment was independently replicated twice with 3 replicate flasks for each drug treatment within each experiment.

### 4.9. Cell Lysate Protein Isolation and Quantification

Cell lysate flowthrough (~700 µL) was mixed with an equal volume of protein precipitator from a NucleoSpin RNA/protein mini kit (Macherey-Nagel 740933, Bethlehem, PA, USA), vortexed, and incubated at room temperature for 10 min. Samples were centrifuged at 11,000× *g* for 5 min, the supernatant discarded, and the pellet washed with 500 µL 50% ethanol. After a second centrifugation (11,000× *g*, 1 min) and removal of supernatant, pellets were air-dried for ~30 min at room temperature and resuspended in 90 µL RIPA buffer (Thermo Fisher Scientific 89900, Waltham, MA, USA). Samples were vortexed, sonicated with a Fisher Scientific Digital Sonic Dismembrator Model 150E for ten 5 sec pulses at 20% amplitude before quantification via Pierce BCA assay (Thermo Fisher Scientific 23227, Waltham, MA, USA), according to the manufacturer’s protocol.

### 4.10. Western Blots

Resolving gels and stacking gels were prepared using standard 37.5:1 acrylamide/bis-acrylamide solutions (Bio-Rad 1610158, Hercules, CA, USA). For the resolving gel, the final mixture contained 9.9% acrylamide/bis, 0.1% SDS (Sigma Aldrich 75746), 0.1% APS (Bio-Rad 1610700, Hercules, CA, USA), and 0.1% TEMED (Sigma Chemical T-9281, St. Louis, MO, USA) in 375 mM Tris-HCl (pH 8.8, Sigma Aldrich T1503, St. Louis, MO, USA). After pouring, the gel surface was overlaid with n-butanol to exclude oxygen and allowed to polymerize. Following polymerization, the butanol overlay was removed, and gels were rinsed with distilled water. The stacking gel contained 4.0% acrylamide/bis, 0.1% SDS, 0.1% APS, and 0.1% TEMED in 125 mM Tris-HCl (pH 6.8). Gels were loaded into a Bio-Rad Mini Protean Tetra System (Bio-Rad 1658001EDU, Hercules, CA, USA); running buffer was 25 mM Tris, 192 mM glycine (VWR Chemicals M103, Radnor, PA, USA), 0.1% *w*/*v* SDS (pH 8.3). Sample volumes corresponding to 5 µg of protein were diluted in RIPA buffer and mixed 1:1 or 1:3 as appropriate with 1× or 4× Laemmli buffer with 5% *v*/*v* β-mercaptoethanol (Sigma Chemical M-7522, St. Louis, MO, USA) to a total volume of 20 µL. After denaturing at 95 °C for 5 min, samples were added to each well. Electrophoresis proceeded for ~60 min at 150 V. Protein was transferred to 0.45 µm nitrocellulose membranes (Thermo Fisher Scientific 88014, Waltham, MA, USA) using a wet-transfer 2-Gel Tetra and Blotting Module (Bio-Rad 1660827EDU, Hercules, CA, USA) after pre-soaking sponges, pads, membrane, and gels in transfer buffer for at least 15 min. Transfer buffer was Tris-Glycine SDS containing 20% *v*/*v* methanol, and transfer occurred over 1 h at 100v. Membranes were rinsed briefly in deionized water prior to staining with 0.1% *w*/*v* Ponceau S (Mallinckrodt Chemicals H312-55, Hazelwood, MO, USA) in 5% acetic acid (Fisher Scientific A38-212, Waltham, MA, USA) to visualize protein bands. After removal of dye with 1× TBST (0.15 M NaCl (Sigma Aldrich S-9625, St. Louis, MO, USA), 0.01 M Tris, 0.05% *v*/*v* Triton X-100 (Sigma Aldrich X100, St. Louis, MO, USA), pH 8.0), membranes were blocked by gently shaking in 1× TBST containing 5% *v*/*v* FCIII for 1 h at room temperature. Membranes were incubated overnight with rocking at 4 °C with primary antibodies diluted 1:200 in 1× TBST plus 5% *v*/*v* FCIII as follows: mouse anti-Histone H3 (1G1, Santa Cruz Biotechnology, sc-517576, Santa Cruz, CA, USA), and mouse anti-α-actin (1A4, Santa Cruz Biotechnology, sc-32251, Santa Cruz, CA, USA). Membranes were washed three times (10 min each) in 1× TBST. Secondary antibody incubation was performed for 1 h at room temperature with gentle shaking using Invitrogen goat anti-mouse IgG (H+L) HRP-conjugated antibody (Thermo Fisher Scientific 31430, Waltham, MA, USA) diluted 1:5000 in 1× TBST plus 5% *v*/*v* FCIII. Membranes were washed six times in 1× TBST (5 min each). For chemiluminescent detection, membranes were incubated for 5-10 min at room temperature with Western Blotting Luminol Reagent (Thermo Fisher Scientific 1863095, Waltham, MA, USA) per manufacturer protocol. Excess reagent was removed, and the membrane was placed on a transparent sheet before imaging with an Azure 600 imaging system (Azure Biosystems, Inc., Dublin, CA, USA) in ChemiBlot imaging mode using autoexposure. Images were inverted prior to quantitative analysis using ImageJ. Fold changes in α-SMA for drug treatments were normalized to Histone H3 relative to the vehicle control from the band density data obtained via ImageJ.

### 4.11. Statistical Analysis

Every experiment was independently replicated at least twice, with 2–4 technical replicates within each experiment. Intracellular ROS (DCFH-DA) data were analyzed using one-way ANOVA followed by Tukey’s HSD post hoc test to compare among treatment groups. Scratch-wound migration data were analyzed using one-way ANOVA followed by Tukey’s HSD post hoc test in GraphPad Prism versions 10 and 11.0.0 (GraphPad Software, San Diego, CA, USA), and treatments not sharing a common letter were considered significantly different (*p* ≤ 0.05). Collagen gel contraction data were analyzed in R (R Foundation for Statistical Computing, Vienna, Austria) using either a one-way ANOVA or, where parametric assumptions were not met, the Kruskal–Wallis nonparametric alternative, followed by Tukey’s HSD or Fisher’s LSD post hoc test, respectively, at a significance level of α = 0.05. Gene expression (qRT-PCR) data were analyzed using one-way ANOVA followed by Dunnett’s post hoc test, comparing each treatment group to the vehicle control. Statistical analyses and figure generation were performed using GraphPad Prism. 

## 5. Conclusions

In conclusion, DHA and *A. annua* tea exhibited the strongest antifibrotic profiles with sustained inhibition of migration, suppression of *ACTA2* and *COL1A1*, upregulation of matrix-remodeling *MMPs*, and reduction of the *ACTA2* protein (α-SMA). *A. afra* showed the strongest antioxidant activity despite lacking ART, while ART was largely inactive against ROS. ART was relatively inactive against all the tested fibrotic parameters. In contrast, DHA was very potent. These findings support further investigation of DHA and *A. annua* as candidate antifibrotic agents. Results also challenge the field to distinguish more carefully among ART-related compounds and argue that whole-plant *Artemisia* preparations should be evaluated as pharmacologically complex mixtures rather than reduced to their ART or other single molecule content. Together, these results suggest that the antifibrotic activity of *Artemisia*-based treatments may extend beyond marker expression to functional inhibition of collagen matrix contraction. In that context, while all treatments inhibited contraction, only *A. annua* was enduring, again suggesting a complex molecular response. However, additional mechanistic studies are needed to identify the responsible compounds and pathways that inhibit collagen production and deposition and with any potential for remodeling the ECM.

## Figures and Tables

**Table 1:** Effect of *Artemisia* teas vs. ART and DHA on ROS reduction in human dermal fibroblasts.

Treatment	Neonatal				Adult			
	Pre-Fibrotic		Pro-Fibrotic		Pre-Fibrotic		Pro-Fibrotic	
	3 DPS	5 DPS	3 DPS	5 DPS	3 DPS	5 DPS	3 DPS	5 DPS
	% ROS Reduction							
ART	10 b	0 b	20 b	2 b	7 b	18 b	17 b	7 b
DHA	0 b	0 b	18 b	−3 b	15 b	12 b	18 b	5 b
*A. annua* tea	25 a	10 a,b	12 b	2 b	53 a	20 a,b	23 b	15 b
*A. afra* tea	35 a	20 a	45 a	17 a	63 a	35 a	33 a	18 a

Data are means of *n* = 3 independent experiments, each with 4 internal replicates. Values within a column not sharing a common letter are significantly different at *p* < 0.05 using one-way ANOVA followed by Tukey’s HSD post hoc test. ROS fluorescence was normalized to metabolic activity and then expressed relative to the corresponding vehicle control. Percent ROS reduction was calculated as 100 − metabolic activity-normalized ROS signal relative to vehicle control. Negative values indicate ROS levels above vehicle control after normalization. DPS = days post-seeding; ART = artemisinin, 50 µM; DHA = dihydroartemisinin, 50 µM; *A. annua* tea = 0.86 mg dry weight (DW)/mL; *A. afra* tea = 0.215 mg DW/mL. Pro-fibrotic conditions included ascorbic acid (ASA) and TGF-β treatment, while pre-fibrotic conditions lacked ASA and TGF-β treatment.

**Table 2:** Fibroblast scratch closure after 24 and 48 h treatment with *Artemisia* teas and pure drugs.

Treatment	Neonatal				Adult			
	Pre-Fibrotic		Pro-Fibrotic		Pre-Fibrotic		Pro-Fibrotic	
	24 h	48 h	24 h	48 h	24 h	48 h	24 h	48 h
	% Scratch Closure							
Vehicle control	76.2 a	97.2 a,b	62.3 a	77.9 b	59.0 a	100.0 a	55.5 a	84.6 a
ART	64.2 a	97.5 a	63.5 a	92.4 a	64.0 a	76.0 a	58.4 a	82.7 a
DHA	13.3 c	22.1 c	8.7 d	13.5 d	6.7 c	9.2 c	6.9 c	13.5 c
*A. annua* tea	18.1 c	28.2 c	21.5 c	27.0 c	11.0 c	12.2 c	8.5 c	17.1 c
*A. afra* tea	46.5 b	75.9 b	42.6 b	84.6 a,b	26.4 b	42.0 b	34.1 b	58.3 b

Values are expressed as mean. Values within a column not sharing a common letter are significantly different (*p* < 0.05, one-way ANOVA with Tukey’s post hoc test). ART = artemisinin, 50 µM; DHA = dihydroartemisinin, 50 µM; *A. annua* tea = 0.86 mg DW/mL; *A. afra* tea = 0.215 mg DW/mL. Pro-fibrotic conditions included ASA and TGF-β treatment, while pre-fibrotic conditions lacked ASA and TGF-β treatment.

**Table 3:** Initial mean contraction rates of fibroblasts 24 h after drug treatments.

Treatment	Pre-Fibrotic	Pro-Fibrotic
	mm^2^/h	
Neonatal		
Vehicle control	−5.5 d	−5.8 b
ART	−5.4 c,d	−5.8 b
DHA	−4.8 b,c	−3.9 a
*A. annua* tea	−4.3 a	−3.3 a
*A. afra* tea	−4.8 a,b	−3.8 a
**Adult**		
Vehicle control	−3.2 d	−4.5 c
ART	−2.9 c,d	−3.6 b,c
DHA	−2.5 b,c	−3.0 a,b
*A. annua* tea	−1.7 a	−1.9 a
*A. afra* tea	−2.2 a,b	−3.3 b,c

Treatment groups with different letters are significantly different at α = 0.05 using either a one-way ANOVA or Kruskal–Wallis Chi-squared nonparametric ANOVA alternative (*n* ≥ 3 per experiment replicate).

**Table 4:** qRT-PCR primers for treated fibroblasts.

Gene	Protein	Forward Primer (5′-3′)	Reverse Primer (5′-3′)
*ACTA2*	α smooth muscle actin (α-SMA)	ACTGCCTTGGTGTGTGACAA	
			CACCATCACCCCCTGATGTC
*CCN2*	Connective tissue growth factor	GTGCCTGCCATTACAACTGTC	
			TCTCACTCTCTGGCTTCATGC
*FN1*	Fibronectin (EDA-FN)	CAGTGGAGTATGTGGTTAGTGTC	
			GTGACCTGAGTGAACTTCAGG
*COL1A1*	Collagen 1 (α1)	GTCAGGCTGGTGTGATGGG	
			GCCTTGTTCACCTCTCTCGC
*MMP1*	Matrix metalloproteinase 1	GCATATCGATGCTGCTCTTTC	
			GATAACCTGGATCCATAGATCGTT
*MMP3*	Matrix metalloproteinase 3	ACCTGACTCGGTTCCGCCTG	
			GTCAGGGGGAGGTCCATAGAGGG
*TANGO1*	Transport and Golgi organization protein 1	TGACCGCAACTCACTACAA	
			AAAGCCATCTCCTTCTGCT
*TIMP1*	Tissue inhibitor of metalloproteinases 1	TGGAAGCCCTTTTCAGAG	
			GGCTGTGAGGAATGCACA
*GAPDH*	Glyceraldehyde 3-phosphate dehydrogenase	GAGTCCACTGGCGTCTTCAC	
			TTCACACCCATGACGAACAT

## Data Availability

The original contributions presented in this study are included in the article/Supplementary Materials. Further inquiries can be directed to the corresponding author.

## Supplementary Materials

The following supporting information can be downloaded at: https://www.mdpi.com/article/10.3390/molecules31162745/s1, Original Western blot images and chromatograms (GCMS and TLC) of the two *Artemisia* species are provided in the Supplemental Materials; Supplemental Figure S1: GCMS *A. annua* and *A. afra*; Supplemental Figure S2 and S3: TLCs; Supplemental original Westerns.

## Abbreviations

ACT	Artemisinin combination therapy
ART	Artemisinin
dART	Deoxyartemisinin
AM	Artemether
AS	Artesunate
ASA	L-ascorbic acid 2-phosphate sesquimagnesium salt hydrate
DCFH-DA	2′7′-dichloroflurorescein diacetate
DHA	Dihydroartemisinin
DPDT	Days post-drug treatment
DPS	Days post-seeding
DPBS	Dulbecco’s phosphate-buffered saline
ECM	Extracellular matrix
FCIII	Fetal Clone III
MMP	Matrix metalloproteinase
PBS	Phosphate-buffered saline
PDT	Post-drug treatment
PS	Post seeding
RFU	Relative fluorescence unit
TGF-β	Transforming growth factor beta
TIMP	Tissue inhibitor of metalloproteinases
α-SMA	Alpha smooth muscle actin
