# Supplementary material for: Artemisia annua and A. afra Teas, Artemisinin, and Dihydroartemisinin Differentially Regulate ROS and Fibrosis-Associated Phenotypes in Human Dermal Fibroblasts
Source: Molecules. 2026 Aug 7;31(16):2745. doi: 10.3390/molecules31162745 (PMC13515305; doi:10.3390/molecules31162745)

Westerns from protein extract from profibrotic neonatal and adult cells post 4d treatment with A. annua, A. afra, ART, or DHA.

There were 4 separately run PCR experiments form which both RNA and total protein were extracted for analyses. The protein was for Western analysis and the experiments with enough extracted protein were analyzed for their replication of translational responses of α-SMA and Histone 3. The following experiments were analyzed and data excerpted and shown in Fig. 5:

- CRL2097e1 and CRL2097e2
- CT1005e2 and CT1005e4

All sample were from pro-fibrotic cells. Responses to the test drugs in prefibrotic cells of neonatal (CRL-2097) cells were previously reported in Weathers et al. Differential Anti-Fibrotic and Remodeling Responses of Human Dermal Fibroblasts to Artemisia sp., Artemisinin, and Its Derivatives. Molecules 2024, 29, 2107. https://doi.org/10.3390/molecules29092107

Reverse images of all generated Western blots from each profibrotic experiment. Yellow highlighted dates are the blots chosen from each independent experiment for illustration and quantitative scanning shown in Fig. 5.

CRL2097e1 5/13

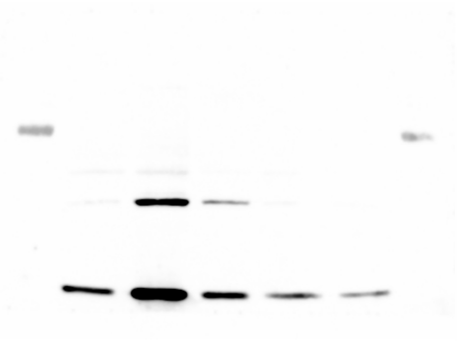

5/27

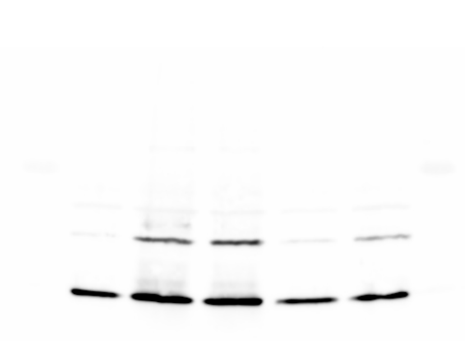

6/4

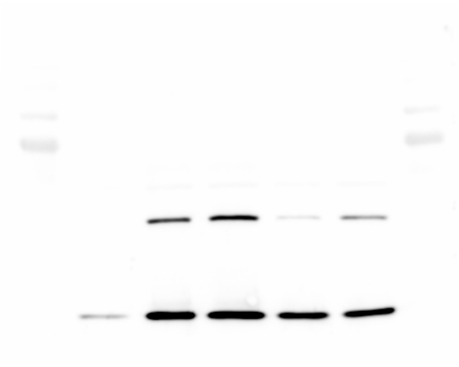

CRL2097e2 5/29 + 6/1

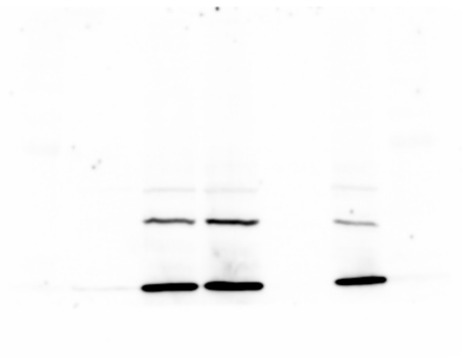

6/5

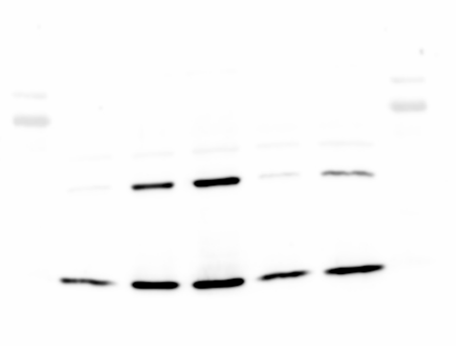

CT1005e2 5/14

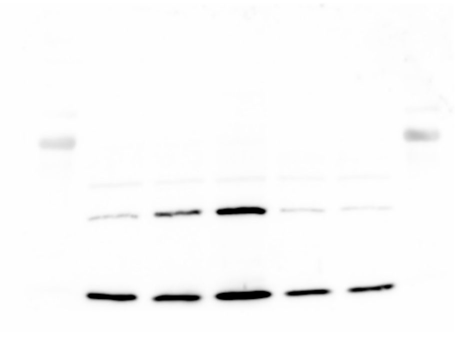

5/27

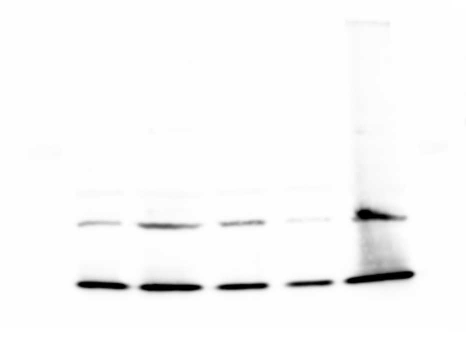

6/4

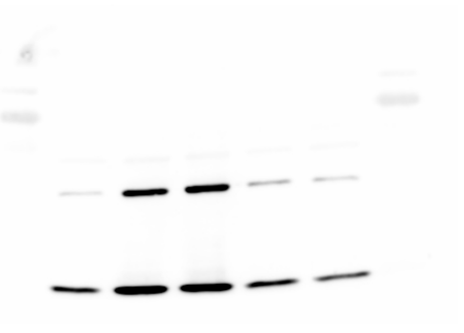

CT1005e4 5/29 + 6/1

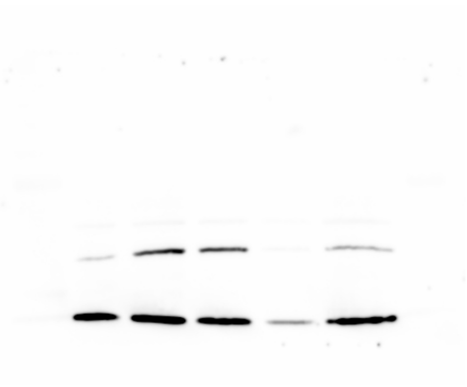

6/5

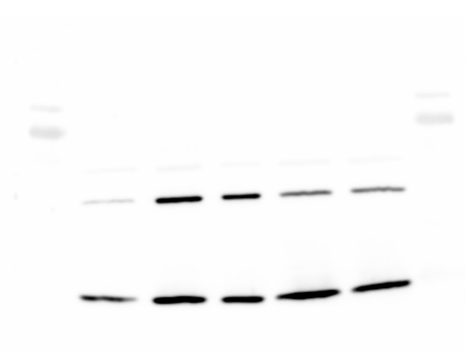

CRL2097 exp1 (profibrotic)

5 µg protein loaded per lane

Histone H3 = 15 kDa, α-actin = 43 kDa

Treatment key:

DHA = 50 µM dihydroartemisinin; ART = 50 µM artemisinin; con = vehicle control; AnT = 8.6% v/v *A. annua* tea (10 g/L); AfT = 2.15% v/v *A. afra* tea (10 g/L)

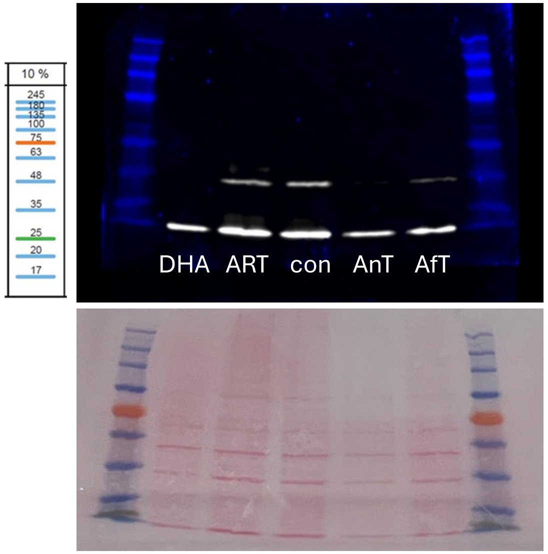

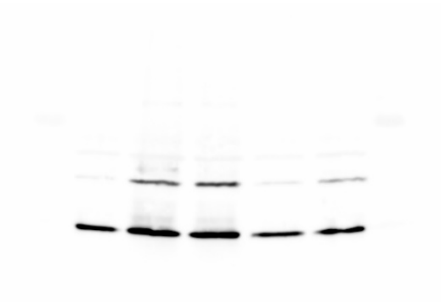

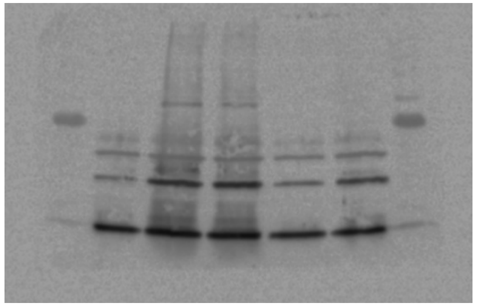

<u>Neonatal</u> <u>- CRL2097 exp2 (profibrotic)</u>

5 μg protein loaded per lane

Histone H3 = 15 kDa, α-actin = 43 kDa

Treatment key:

DHA = 50 μM dihydroartemisinin; ART = 50 μM artemisinin; con = vehicle control; AnT = 8.6% v/v *A. annua* tea (10 g/L); AfT = 2.15% v/v *A. afra* tea (10 g/L)

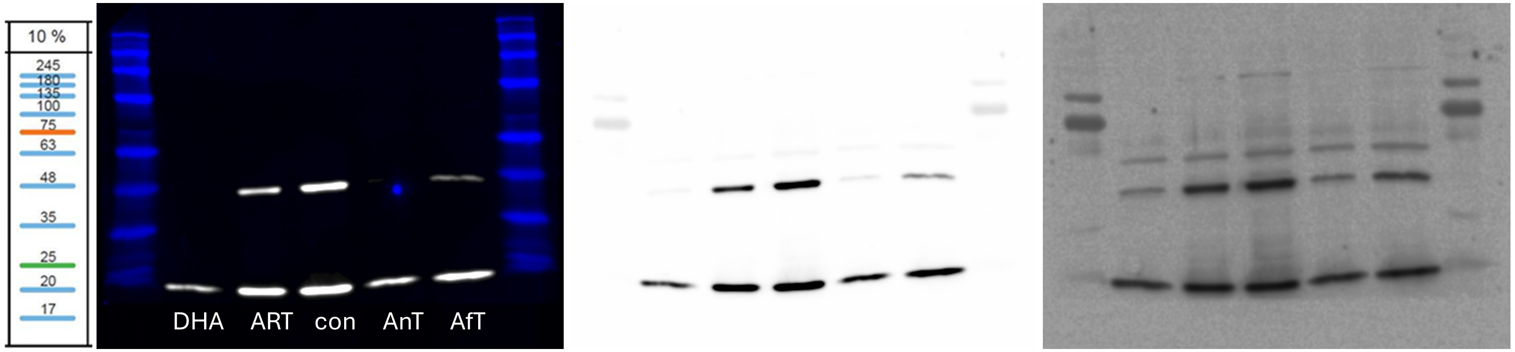

Adult CT1005 exp2 (profibrotic)

5 µg protein loaded per lane

Histone H3 = 15 kDa, α-actin = 43 kDa

Treatment key:

DHA = 50 µM dihydroartemisinin; ART = 50 µM artemisinin; con = vehicle control; AnT = 8.6% v/v *A. annua* tea (10 g/L); AfT = 2.15% v/v *A. afra* tea (10 g/L)

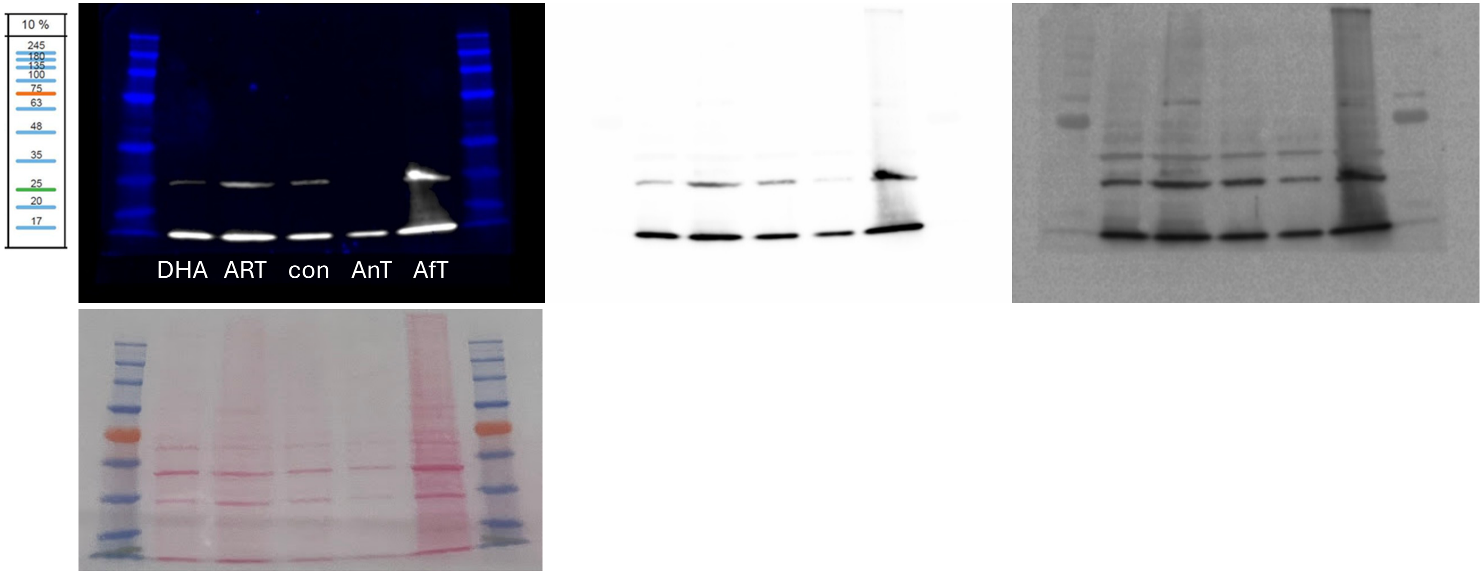

Adult CT1005 exp4 (profibrotic)

5 µg protein loaded per lane

Histone H3 = 15 kDa, α-actin = 43 kDa

Treatment key:

DHA = 50 µM dihydroartemisinin; ART = 50 µM artemisinin; con = vehicle control; AnT = 8.6% v/v *A. annua* tea (10 g/L); AfT = 2.15% v/v *A. afra* tea (10 g/L)

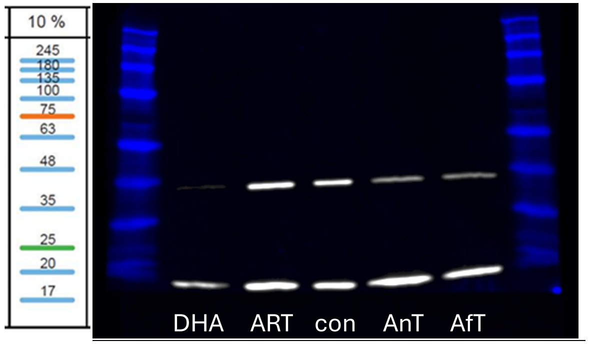

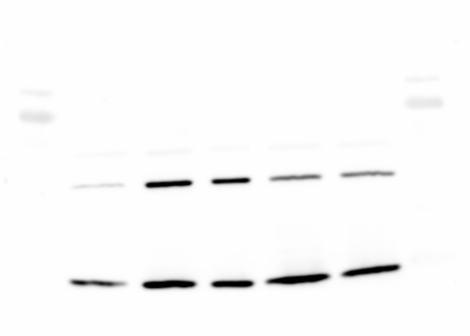

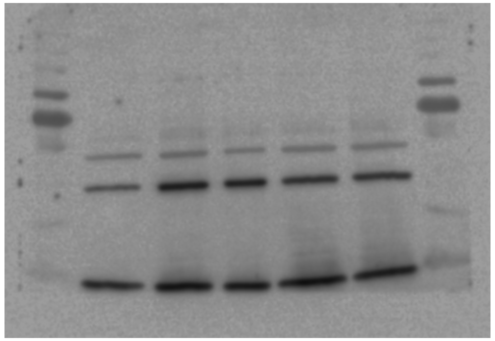

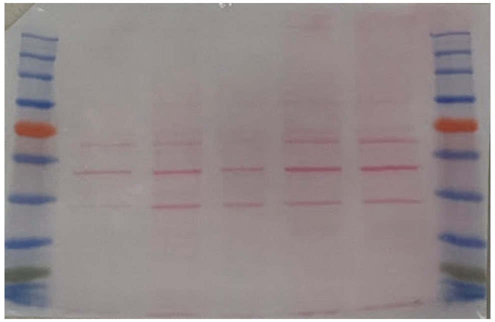

<u>Adult</u> <u>CT1005 exp2 (profibrotic)</u>

5 µg protein loaded per lane

Histone H3 = 15 kDa, α-actin = 43 kDa

Treatment key:

DHA = 50 µM dihydroartemisinin; ART = 50 µM artemisinin; con = vehicle control; AnT = 8.6% v/v *A. annua* tea (10 g/L); AfT = 2.15% v/v *A. afra* tea (10 g/L)

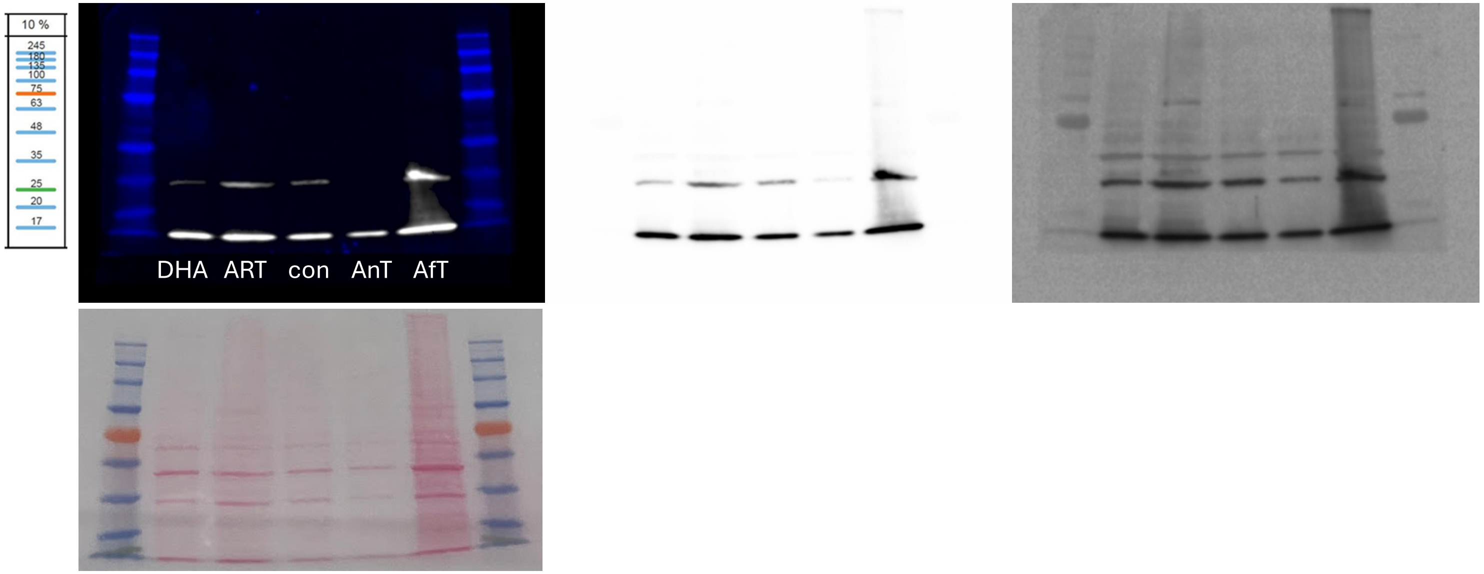

Supplement: Supplementary file 1 [file molecules-31-02745-s001.zip › Supplemental - original Westerns.pdf]
