# Supplementary material for: Artemisia annua and A. afra Teas, Artemisinin, and Dihydroartemisinin Differentially Regulate ROS and Fibrosis-Associated Phenotypes in Human Dermal Fibroblasts
Source: Molecules. 2026 Aug 7;31(16):2745. doi: 10.3390/molecules31162745 (PMC13515305; doi:10.3390/molecules31162745)
Supplement: Supplementary file 1 [file molecules-31-02745-s001.zip › Supplemental Figure S1 GCMS A annua and A afra.pdf]

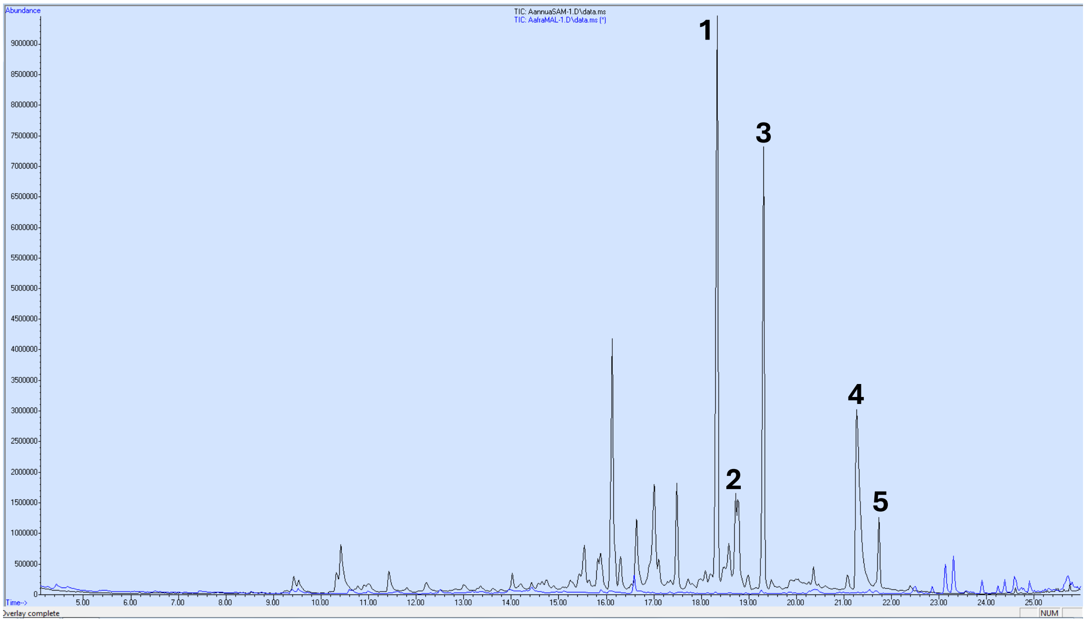

Figure S1. GCMS chromatogram of methylene chloride extracts of *Artemisia annua* Artecinua™ (black) overlayed with *Artemisia afra* (blue). Peak 1 = deoxyartemisinin; peak 2 = arteannuin B; peak 3 = artemisinin breakdown product #1; peak 4 = artemisinin breakdown product #2; peak 5 = artemisinin

Artemisia annua:
Data Path : D:\MassHunter\GCMS\1\data\eeyore\AFRA-ANNUA-CHROMATOGRAMS\
Data File : AannuaSAM-1.D
Acq On : 14 Jul 2026 17:28
Operator : MJT
Sample :
Misc : 0.5mg SAM in 50 uL MeCl2 B#1.Sh6.01.15.20 capsules
ALS Vial : 5 Sample Multiplier: 1

Search Libraries: D:\DATABASE\AN_METABOLITES.L Minimum Quality: 0
D:\DATABASE\NIST08.L Minimum Quality: 0

Unknown Spectrum: Apex
Integration Events: ChemStation Integrator - autoint1.e

Pk#	RT	Area%	Library/ID	Ref#	CAS#	Qual
1	9.430	0.71	D:\DATABASE\AN_METABOLITES.L			
			No matches found			
2	9.542	0.59	D:\DATABASE\AN_METABOLITES.L			
			thujone	39	000000-00-0	15
			alpha-pinene	21	000000-00-0	7
			nerolidol	33	1000285-43-6	7
3	10.334	0.60	D:\DATABASE\AN_METABOLITES.L			
			No matches found			
4	10.422	2.67	D:\DATABASE\AN_METABOLITES.L			
			No matches found			
5	11.437	0.98	D:\DATABASE\AN_METABOLITES.L			
			DHA	9	000000-00-0	7
6	12.227	0.67	D:\DATABASE\AN_METABOLITES.L			
			No matches found			
7	14.026	0.69	D:\DATABASE\AN_METABOLITES.L			
			DHA	9	000000-00-0	12
8	15.254	1.06	D:\DATABASE\AN_METABOLITES.L			
			No matches found			
9	15.440	0.74	D:\DATABASE\AN_METABOLITES.L			
			No matches found			
10	15.543	1.89	D:\DATABASE\AN_METABOLITES.L			
			No matches found			

11 15.832 1.15 D:\DATABASE\AN_METABOLITES.L
No matches found

12 15.890 1.68 D:\DATABASE\AN_METABOLITES.L
thujone 49 000000-00-0 10
thujone 39 000000-00-0 10

13 16.125 8.90 D:\DATABASE\AN_METABOLITES.L
No matches found

14 16.304 1.31 D:\DATABASE\AN_METABOLITES.L
No matches found

15 16.647 3.19 D:\DATABASE\AN_METABOLITES.L
No matches found

16 17.019 6.22 D:\DATABASE\AN_METABOLITES.L
No matches found

17 17.105 1.33 D:\DATABASE\AN_METABOLITES.L
No matches found

18 17.485 3.60 D:\DATABASE\AN_METABOLITES.L
No matches found

19 17.729 0.58 D:\DATABASE\AN_METABOLITES.L
deoxyqinghaosu 20 1000126-03-6 14
deoxyAN-2 10 000000-00-0 7

20 17.994 0.69 D:\DATABASE\AN_METABOLITES.L
No matches found

21 18.090 0.92 D:\DATABASE\AN_METABOLITES.L
No matches found

22 18.204 0.70 D:\DATABASE\AN_METABOLITES.L
No matches found

23 18.341 19.04 D:\DATABASE\AN_METABOLITES.L
deoxyAN-2 10 000000-00-0 99
NIST artemisia ketone 47 000546-49-6 64
deoxyqinghaosu 20 1000126-03-6 56

24 18.483 1.21 D:\DATABASE\AN_METABOLITES.L
DeoxyAN 6 000000-00-0 9

25 18.588 2.13 D:\DATABASE\AN_METABOLITES.L

DeoxyAN 6 000000-00-0 7

26 18.733 3.19 D:\DATABASE\AN_METABOLITES.L
Arteannuin B 3 000000-00-0 46

27 18.779 2.98 D:\DATABASE\AN_METABOLITES.L
No matches found

28 18.986 0.61 D:\DATABASE\AN_METABOLITES.L
Artemisinin (NIST Library) 4 000000-00-0 47

29 19.319 13.31 D:\DATABASE\AN_METABOLITES.L
Artemisinin (3 peaks) 2 000000-00-0 98
deoxyqinghaosu 20 1000126-03-6 2

30 19.479 0.57 D:\DATABASE\AN_METABOLITES.L
DeoxyAN 6 000000-00-0 10

31 19.880 0.71 D:\DATABASE\AN_METABOLITES.L
deoxyAN-2 10 000000-00-0 7
deoxyqinghaosu 20 1000126-03-6 4
NIST artemisia ketone 47 000546-49-6 4

32 20.361 0.88 D:\DATABASE\AN_METABOLITES.L
NIST artemisia ketone 47 000546-49-6 25

33 21.083 0.58 D:\DATABASE\AN_METABOLITES.L
No matches found

34 21.278 11.40 D:\DATABASE\AN_METABOLITES.L
ANunknownNEW 40 000000-00-0 94
AN unknown peak 11 000000-00-0 89

35 21.740 2.51 D:\DATABASE\AN_METABOLITES.L
Artemisinin (NIST Library) 4 000000-00-0 95
ANnistNEW 41 000000-00-0 11

Artemisia afra:
Data Path : D:\MassHunter\GCMS\1\data\eeyore\AFRA-ANNUA-CHROMATOGRAMS\
Data File : AafraMAL-1.D
Acq On : 14 Jul 2026 17:58
Operator : MJT
Sample :
Misc : 0.5mg MAL in 50 uL MeCl2 B#1.SB.AF.Rb7.04.02.21
ALS Vial : 6 Sample Multiplier: 1

Search Libraries: D:\DATABASE\AN_METABOLITES.L Minimum Quality: 0
D:\DATABASE\NIST08.L Minimum Quality: 0

Unknown Spectrum: Apex
Integration Events: ChemStation Integrator - autoint1.e

Pk# RT Area% Library/ID Ref# CAS# Qual

---

1 16.594 10.84 D:\DATABASE\AN_METABOLITES.L
No matches found

2 23.142 20.19 D:\DATABASE\AN_METABOLITES.L
No matches found

3 23.311 26.33 D:\DATABASE\AN_METABOLITES.L
No matches found

4 23.913 8.53 D:\DATABASE\AN_METABOLITES.L
No matches found

5 24.385 5.98 D:\DATABASE\AN_METABOLITES.L
No matches found

6 24.589 14.77 D:\DATABASE\AN_METABOLITES.L
No matches found

7 24.915 5.28 D:\DATABASE\AN_METABOLITES.L
No matches found

8 25.716 8.08 D:\DATABASE\AN_METABOLITES.L
No matches found
