# Supplementary material for: Artemisia annua and A. afra Teas, Artemisinin, and Dihydroartemisinin Differentially Regulate ROS and Fibrosis-Associated Phenotypes in Human Dermal Fibroblasts
Source: Molecules. 2026 Aug 7;31(16):2745. doi: 10.3390/molecules31162745 (PMC13515305; doi:10.3390/molecules31162745)
Supplement: Supplementary file 1 [file molecules-31-02745-s001.zip › Supplemental Figure S2 & S3 TLCs 2026.pdf]

Artemisinin & other organic compounds (anisaldehyde stain)

Equivalent of 0.5 mg DW of extracted leaf per spot. The method is: 25 mg of dried leaves are extracted in 4 mL dichloromethane (DCM). Plastic-backed silica gel 60 F_{254} plates are used as the stationary phase. The mobile phase is a 5:11:4 mixture of methylene chloride, hexane, and acetone, plus 0.5% v/v glacial acetic acid. Artemisinin (ART) and various other organic compounds are visualized via *p*-anisaldehyde reagent (1 mL glacial acetic acid, 2.5 mL sulfuric acid, 75 mL ethanol, and 2 mL p-anisaldehyde), where ART can be seen as a deep pink spot (Figure S1).

Left to right: (S = *Artemisia annua* SAM cv; M = *A. afra* MAL cv; 1, 2, 3 refer to replicate extracts)

A. annua SAM DCM extract, SAM tea, SAM tea extract; *A. afra* MAL DCM extract, MAL tea, MAL tea extract; *A. annua* SAM DCM extract, SAM tea extract; 5 μg artemisinin standard; *A. afra* MAL DCM extract, MAL tea extract; *A. annua* SAM DCM extract, SAM tea, SAM tea extract; *A. afra* MAL DCM extract, MAL tea, MAL tea extract

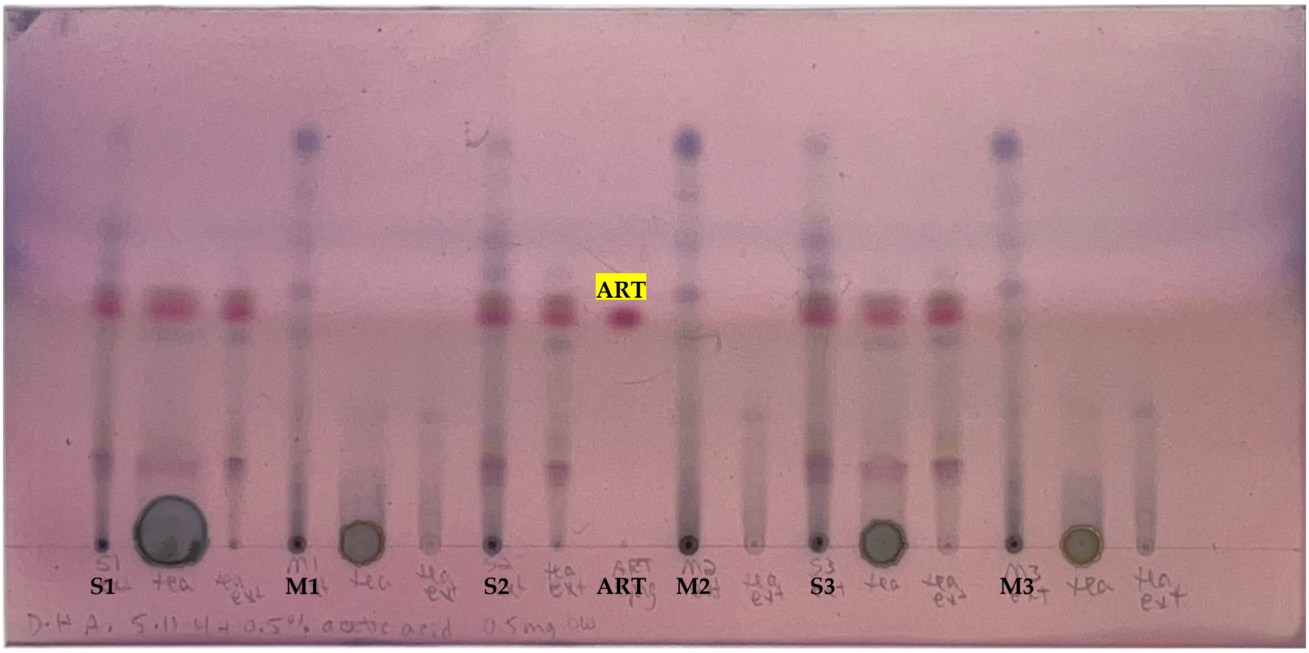

Figure S2. Triplicate samples of 0.5 g DW equivalent of *A. annua* and *A. afra*, dichloromethane extracts of dried leaves, unextracted tea infusion (10 g DW/L), and DCM extract of prepared tea infusion stained with *p*-anisaldehyde reagent.

General flavonoids (DPBA stain)

Equivalent of 0.5 mg DW of extracted leaf per spot. The method is: 25 mg of dried leaves are extracted in 4 mL methylene chloride. Plastic-backed silica gel 60 F_{254} plates are used as the stationary phase. Mobile phase is a 10:8:2:1 mixture of toluene, ethyl acetate, formic acid, and water. General flavonoids are visualized using 0.5% (weight/volume, w/v) 2-aminoethyl diphenylborinate (DPBA) in methanol and viewed under UV light.

Left to right: (S = *Artemisia annua* SAM cv; M = *A. afra* MAL cv; 1, 2, 3 refer to replicate extracts)

A. annua SAM DCM extract, SAM tea, SAM tea extract; *A. afra* MAL DCM extract, MAL tea, MAL tea extract; *A. annua* SAM DCM extract, SAM tea extract; *A. afra* MAL DCM extract, MAL tea extract; *A. annua* SAM DCM extract, SAM tea, SAM tea extract; *A. afra* MAL DCM extract, MAL tea, MAL tea extract

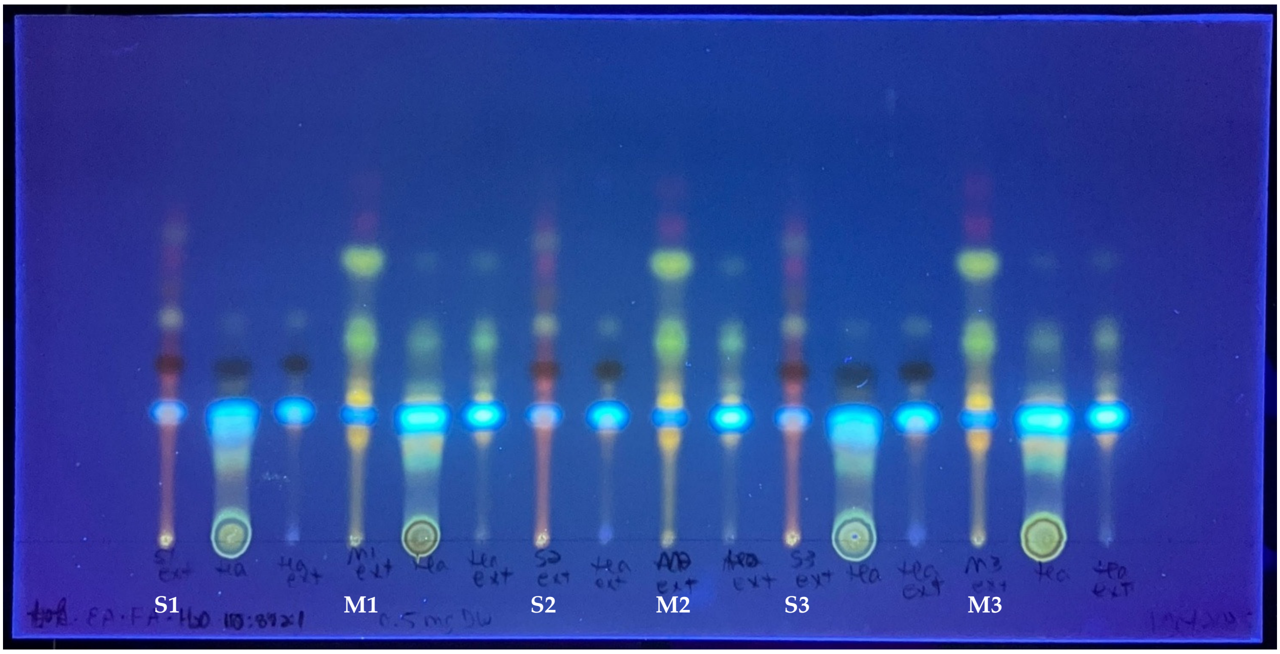

Figure S3. Triplicate samples of 0.5 g DW equivalent of *A. annua* and *A. afra*, dichloromethane extracts of dried leaves, unextracted tea infusion (10 g DW/L), and DCM extract of prepared tea infusion stained with 2-aminoethyl diphenylborinate (DBPA).
